# Essential genes from genome-wide screenings as a resource for neuropsychiatric disorders gene discovery

**DOI:** 10.1038/s41398-021-01447-y

**Published:** 2021-05-25

**Authors:** Wei Zhang, Joao Quevedo, Gabriel R. Fries

**Affiliations:** 1 Translational Psychiatry Program, Louis A. Faillace, MD, Department of Psychiatry & Behavioral Sciences, McGovern Medical School, The University of Texas Health Science Center at Houston, 1941 East Rd, 77054 Houston, TX USA; 2 Neuroscience Graduate Program, The University of Texas MD Anderson Cancer Center UTHealth Graduate School of Biomedical Sciences, Houston, TX USA; 3 Center of Excellence in Mood Disorders, Louis A. Faillace, MD, Department of Psychiatry & Behavioral Sciences, The University of Texas Health Science Center at Houston, 1941 East Rd, 77054 Houston, TX USA; 4 Translational Psychiatry Laboratory, Graduate Program in Health Sciences, University of Southern Santa Catarina (UNESC), Criciúma, SC Brazil; 5 Center for Precision Health, School of Biomedical Informatics, The University of Texas Health Science Center at Houston, 7000 Fannin St, 77030 Houston, TX USA

**Keywords:** Genomics, Psychiatric disorders, Biomarkers

## Abstract

Genome-wide screenings of “essential genes”, i.e., genes required for an organism or cell survival, have been traditionally conducted in vitro in cancer cell lines, limiting the translation of results to other tissues and non-cancerous cells. Recently, an in vivo screening was conducted in adult mouse striatum tissue, providing the first genome-wide dataset of essential genes in neuronal cells. Here, we aim to investigate the role of essential genes in brain development and disease risk with a comprehensive set of bioinformatics tools, including integration with transcriptomic data from developing human brain, publicly available data from genome-wide association studies, de novo mutation datasets for different neuropsychiatric disorders, and case–control transcriptomic data from postmortem brain tissues. For the first time, we found that the expression of neuronal essential genes (NEGs) increases before birth during the early development of human brain and maintains a relatively high expression after birth. On the contrary, common essential genes from cancer cell line screenings (ACEGs) tend to be expressed at high levels during development but quickly drop after birth. Both gene sets were enriched in neurodevelopmental disorders, but only NEGs were robustly associated with neuropsychiatric disorders risk genes. Finally, NEGs were more likely to show differential expression in the brains of neuropsychiatric disorders patients than ACEGs. Overall, genome-wide central nervous system screening of essential genes can provide new insights into neuropsychiatric diseases.

## Introduction

The human brain has often been considered the reason why humans outstand from other animals, having the largest ratio among body size, consuming more energy than any other organ^1^, and containing around 100 billion neurons and ten times more glial cells. Because neurons are usually found in a quiescent state in the adult nervous system, they are usually regarded as post-mitotic cells and are more likely to undergo apoptosis in response to cell cycle reactivation^2^. Many types of human diseases are related to neuronal function and survival, including neurodevelopmental disorders (NDDs)^3^, neurodegenerative diseases, and psychiatric disorders. NDDs, which have been associated with abnormal brain development^3^, include autism spectrum disorder (ASD), intellectual disability (ID), communication disorders, attention deficit/hyperactivity disorder, among others, with symptoms encompassing impairments in communication, cognition, and behavior. Neurodegenerative diseases include Alzheimer’s disease (AD), Parkinson’s disease (PD), Huntington’s diseases (HD), and amyotrophic lateral sclerosis (ALS), and are typically characterized by progressive damage to cells of patients’ nervous system that can result in impairments in mobility, coordination, sensation, and cognition^4^. Finally, psychiatric disorders are conditions that may affect the mood, thinking, feeling, and behavior of patients, and include major depressive disorder, bipolar disorder (BD), and schizophrenia (SCZ)^5^, among other conditions.

“Essential genes” are genes required for an organism or cell survival, and they have been directly assessed and identified in a number of species using high-throughput cellular and animal models^6^, such as yeast^7^ and mouse^8^. The systematic, genome-wide screening of essential genes in different organisms has enhanced our understanding of the molecular bases of many biological processes^9,10^. For example, Patel et al. used genome-wide screening to identify essential genes with key functions in antigen presentation and interferon-γ signaling for the development of cancer immunotherapy^11^. Essential genes can be easily investigated at the cellular level using cancer cell line knock-out or knock-down screenings based on gene-trapping, RNA interference (RNAi), or gene-editing such as transcription activator-like effector nucleases or CRISPR-Cas9. Project Achilles is the largest project that aims to characterize 1000 cancer cell lines for the systematic identification of essential genes^12^. In their efforts, they have identified a list of genes that are universally essential for all studied cell lines, named “Achilles common essentials genes” (ACEGs).

While the original purpose of essential gene screenings in cancer cell lines is to study tumor biology, their results may reflect the general requirement of genes in all tissues. Accordingly, their identification may provide insights into other disorders or processes related to cell viability, including neurodegenerative disorders, hallmark of which is the progressive death of specific sets of neurons^4^. However, due to differences between cancer cell lines and in viv*o* healthy living cells, in vitro cell line screenings have limitations when applied to the neuropsychiatric field. Most brain tumors do not come from neurons, but from glial cells or other nonneuronal cells in the central nervous system (CNS). Even neuronal tumors^13^, a rare group of brain tumors consisting of abnormal neurons, have significant differences from the in vivo, post-mitotic neurons. Recently, Wertz et al.^14^ published a paper using, for the first time, a genome-wide in vivo screening in adult mice brain tissue to identify neuronal-specific essential genes (NEGs), which can theoretically be a better source of essential genes for neurological and psychiatric studies. To that aim, the authors developed a method in which they initially introduced genome-wide pooled screening libraries (for RNAi- and CRISPR-based screenings) that targeted most of the protein-coding genes into adult mouse striata, followed by tissue dissection and genomic DNA sequencing for library preparation. The rationale was that target genes underrepresented in the tissue relative to the input libraries represented genes for which knock-down or knock-out lead to striatum toxicity because they lead to cell death. Interestingly, this genome-wide CNS screening found that neurons are not only sensitive to perturbations to synaptic processes, but also autophagy, proteostasis, mRNA processing, and mitochondrial function. The study also found that some of these NEGs were transcriptionally downregulated in two HD mouse models, suggesting their usefulness for the study of brain-relevant diseases. Here we compared this NEG set with the common essential gene set identified in cancer cell lines. Our results show that these two gene sets are in different co-expression networks in the developing human brain. Moreover, while they both undergo de novo mutations in neuropsychiatric patients, we found that only NEGs are directly related to neuropsychiatric disorders.

## Materials and methods

### Datasets and GEO Data

We have used multiple publicly available datasets for this study. The NEG list from in vivo screening was obtained from the supplementary data of the original paper^14^, and the common essential gene list was downloaded from Project Achilles^12^ website (https://depmap.org/portal/download/). This dataset comprises viability data on 17,634 genes in 485 cell lines. The schizophrenia case–control data were obtained from the CommonMind Consortium (DESeq2 analyzed data provided by the authors at https://www.synapse.org/#!Synapse:syn5609493)^15^. GWAS summary statistics data were downloaded from GWAS Catalog^16^ (https://www.ebi.ac.uk/gwas/). Finally, de novo mutation data were downloaded from de novo-db v.1.6.1 (http://denovo-db.gs.washington.edu/denovo-db/Download.jsp)^17^. The mutations specific for each disorder were extracted from the whole data, and any data with missing gene ID or patient ID were discarded.

The following Gene Expression Omnibus (GEO) data were also downloaded for use in this study: (1) GSE95587: RNA-seq from fusiform gyrus of 84 AD patients and 33 neurologically normal post-mortem tissues (ages 60–103, 51 females, 66 males)^18^; (2) GSE122649: RNA-seq analysis of postmortem cortex from 25 ALS patients and 12 control individuals without neurological disorders (ages 54-81, 16 females, 21 males)^19^; (3) GSE64018: RNA-seq from BA41 region of 12 ASD patients and 12 healthy controls (ages 15–60, 6 females, 18 males)^20^; (4) GSE64810: RNA-seq from post-mortem BA9 brain tissue of 49 neurologically normal individuals and 20 HD patients (ages 36–106, 29 females, 40 males)^21^; and (5) GSE68719: RNA-seq from post-mortem dorsolateral prefrontal cortex of 30 PD patients versus 29 controls (ages 46–97, all males)^22^.

### RNA-Seq data analysis

Raw fastq files (listed above) were downloaded from the GEO website. FastQC^23^ v0.11.9 was used for quality control of the sequencing data, and Trimmomatic^24^ v0.36 was used to trim out low quality reads. Reads were mapped to hg38 human genome using STAR^25^ v2.7.7, and the abundance of genes was calculated using HTSeq^26^ v0.13.5. DESeq2 R package^27^ was used to identify differentially expressed (DE) genes using library size, RNA integrity number, and postmortem interval as covariates. The obtained p-values were used for downstream two-sided Wilcoxon rank-sum tests. The schematic workflow for these analyses is shown in Supplementary Fig. 1.

### Cohen’s *d*

We calculated the Cohen’ s *d* using the formulas below, with Y representing the –log10(*p* value) from DEseq2 results for the NEGs and X representing the –log10(*p* value) from DEseq2 results for the ACEGs. Because the sizes of the two groups were not equal, we used pooled standard deviation for all calculations.

$${\mathrm{d}} = \frac{{\bar Y - \bar X}}{s}$$

$$s = \sqrt {\frac{{\left( {n_X - 1} \right)s_X^2 + \left( {n_Y - 1} \right)s_Y^2}}{{n_X + n_Y - 2}}}$$

### Analyses of general features of genes

Gene probabilities of mutations (all types) were obtained from Samocha et al.^28^ These analyses calculate the per-gene probability of mutation by summing up the probability of de novo mutations of each base in the coding region. The probability of loss-of-function intolerance (pLI) of genes was estimated based on Lek et al.^29^ using an expectation-maximization algorithm, calculated from exome DNA sequencing data from 60,706 individuals of diverse ethnic background. Gene-level Integrated Metric of Negative Selection (GIMS) scores, an indicator for the degree of negative selection in molecular evolution, were obtained according to Sampson et al.^30^

### GWAS data analysis

The downloaded summary statistics data were analyzed with Multi-marker Analysis of GenoMic Annotation (MAGMA)^31^. MAGMA uses a multiple regression framework that converts genetic marker data to gene-level using mean SNP *p* values, and after calculating for linkage disequilibrium, tests the enrichment between each trait and genes in a given gene set compared to genes outside the gene set. We downloaded GWAS summary statistics and used MAGMA to map SNPs onto genes and compute gene *p* values. Finally, we performed a competitive gene set analysis to calculate if certain gene sets were enriched in the genes with significant SNPs.

### GO enrichment analysis

GO analysis was conducted using the WEB-based GEne SeT AnaLysis Toolkit (WebGestalt^32^; http://www.webgestalt.org/). The Over-Representation Analysis method was employed to test against the gene-ontology database (http://geneontology.org/) with Benjamini–Hochberg method for multiple test adjustment (false discovery rate threshold of 0.05). Enrichment scores were calculated to reflect the degree to which a set of genes was overrepresented at the extremes (top or bottom) of the ranked reference sets^33^.

### WGCNA

Co-expression modules were created with the weighted gene co-expression network analysis^34^ (WGCNA) package using BrainSpan data^35^, which covers full period of brain development and all available brain regions (5.7 PCW—70 years of age; 1340 samples). An adjacency matrix was created by computing the correlation between the expression levels of each gene with every other gene using signed biweight correlations; a soft threshold of power of 15 was used to achieve scale free topology (Supplementary Fig. 2). The adjacency matrix was transformed into a topological overlap matrix (TOM), and the network dendrogram (Supplementary Fig. 3) was generated from the TOM dissimilarity matrix (1-TOM). The “Dynamic Hybrid” method was employed with standard parameters (i.e., pamStage = TRUE, minimum module size = 30, deepSplit = 2, pamRespectsDendro=FALSE), and modules with ME correlations >0.9 were merged. Twenty-two modules were generated.

### Tissue expression enrichment analysis

We used the deTS^36^ R package, which has a built-in GTEx data as reference, to query our two lists of genes with the Fisher’s Exact Test. Cell-type Specific Expression Analysis (CSEA^37^), a web-based tool which accepts gene lists as input and returns an enrichment analysis of their expression across the different cell types and regions, was also performed. Fisher’s exact tests and Benjamini-Hochberg correction were implemented to identify a candidate gene list that overlaps with genes whose expressions were enriched in a particular cell type or region.

## Results

### GO analysis shows that NEGs are more specifically enriched in neuronal terms

**Fig. 1:**
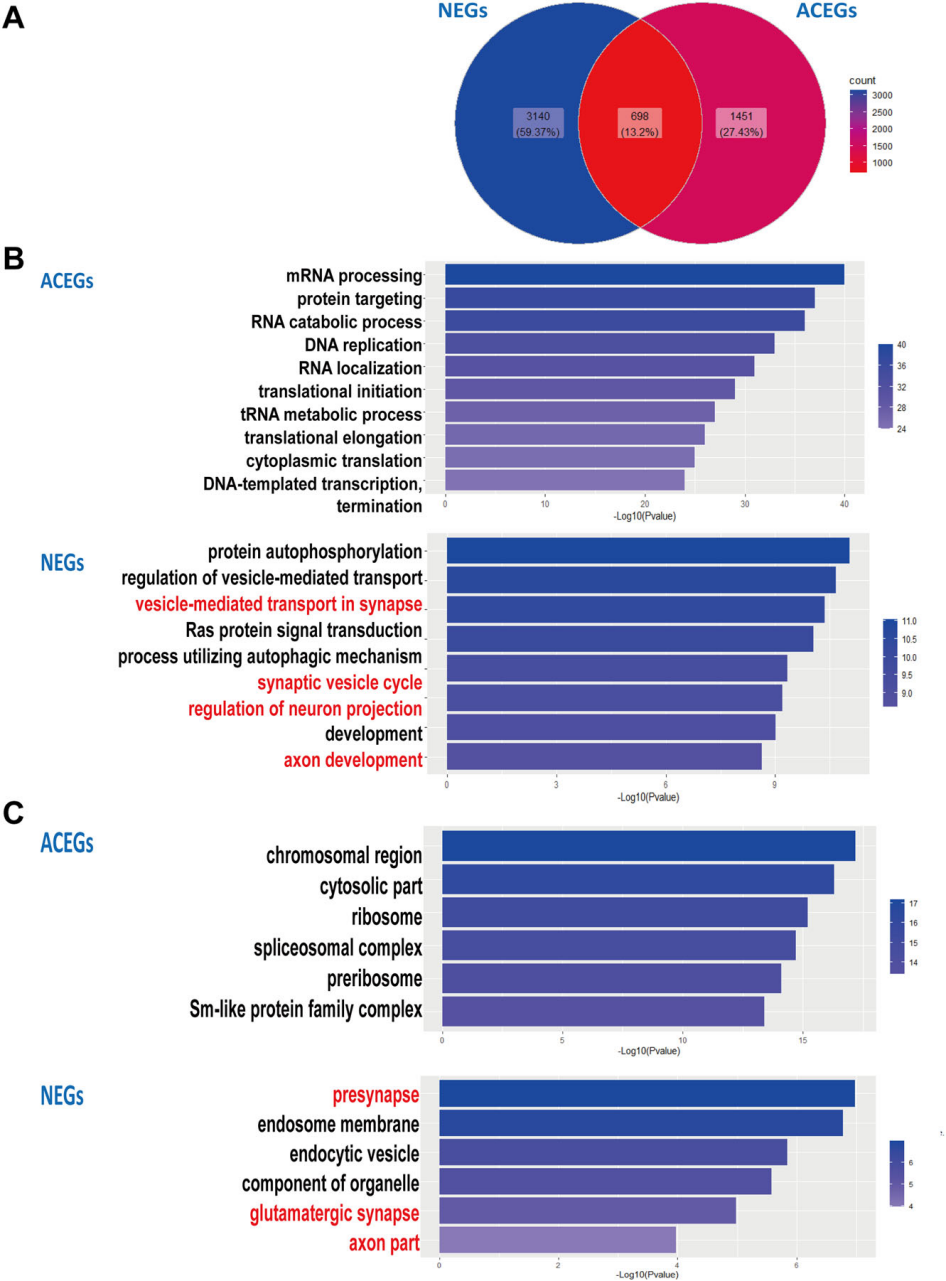
Gene ontology (GO) analysis of essential genes. **a** Venn diagram shows overlapping of Neuronal Essential Genes (NEGs) and Achilles Project-specific essential genes (ACEGs). **b** Top biological process GO terms enriched in NEGs and ACEGs. **c** Top cellular component GO terms enriched in NEGs and ACEGs.

We initially downloaded the neuronal essential genes list^14^ and mapped the gene symbols from the mouse gene list into human orthologues using the R package biomaRt^38^, obtaining 3838 neuronal essential human genes. Then, we obtained the common essential gene list containing 2149 genes from Project Achilles (https://depmap.org/portal/download/). There were 698 genes that were common between these two datasets (Fig. 1A), supposedly being essential for both cancer cell lines and post-mitotic neuronal survival. In theory, the non-common genes in both lists should have different biological meanings. We named the 3140 neuronal-specific essential gene as “NEGs” and the 1451 Achilles Project-specific essential genes as “ACEGs”. We then ran gene set enrichment analysis using Gene Ontology (GO) annotations with the non-common gene lists in both datasets using WebGestalt^39^. As expected, the results show that NEGs are more likely to be enriched in neuron-specific biological process, such as GO:0099003 (vesicle-mediated transport in synapse), GO:0099504 (synaptic vesicle cycle), GO:0010975 (regulation of neuron projection development), and GO:0061564 (axon development). However, ACEGs are highly enriched in general biological functions, such as GO:0006397 (mRNA processing), GO:0008380 (RNA splicing), and GO:1901987 (regulation of cell cycle phase transition) (Fig. 1B). Cellular component analysis showed that NEGs are primarily enriched in neural components, such as synapse and axons (Fig. 1C). The complete list of the GO terms with FDR less than 0.05 is shown in Supplementary Tables 1–6. Overall, these initial characterization findings confirm that while NEGs are more involved in neuronal function and structures, ACEGs are rather involved in general biological activities relevant to multiple tissues.

### NEGs are more likely to exhibit differential expression in brain tissue in neuropsychiatric disorders compared to ACEGs

**Fig. 2:**
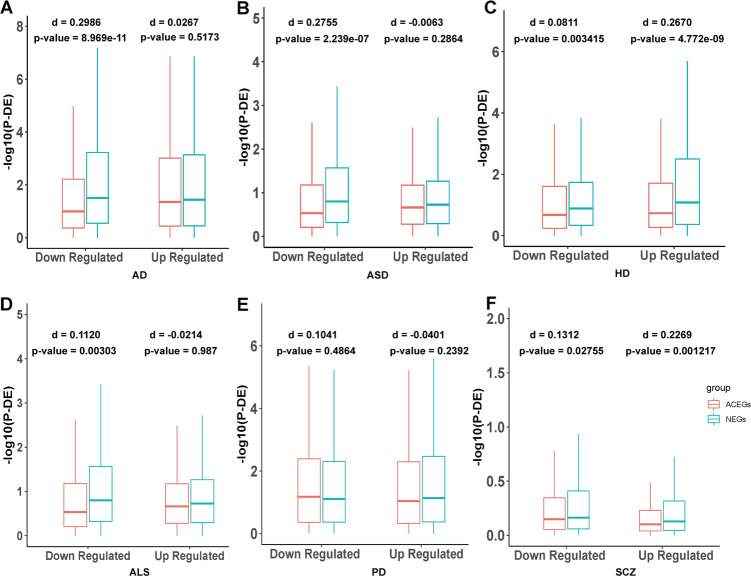
Differential expression (DE) among control and neuropsychiatric disorders in brain tissue transcriptome data. **a** DE *p* value of Neuronal Essential Genes (NEGs) and Achilles Project-specific essential genes (ACEGs) in GSE95587 (fusiform gyrus tissue sections from Alzheimer’s disease (AD) patients and control). **b** DE *p* value of NEGs and ACEGs in GSE64018 (brain cortex from Autism spectrum disorder (ASD) patients and controls). **c** DE *p* value of NEGs and ACEGs in GSE64810 (BA9 tissue from Huntington’s Disease (HD) patients and controls). **d** DE *p* value of NEGs and ACEGs in GSE122649 (brain cortex from Amyotrophic Lateral Sclerosis (ALS) patients and controls). **e** DE *p* value of NEGs and ACEGs in GSE68719 (BA9 tissue from Parkinson’s disease (PD) patients and controls). **f** DE *p* value of NEGs and ACEGs in CommondMind Consortium data (brain cortex from schizophrenia (SCZ) patients and controls).

RNA-seq to detect differentially expressed genes between patients and controls is a very common approach in biomedical research. We identified 6 datasets in the GEO database with RNA-seq from brain tissue of several case–control studies, including AD, ALS, ASD, HD, PD, and SCZ. We initially downloaded the raw gene expression data and then used the R package DESeq2 to normalize them, or downloaded the DESeq2 normalized data directly, followed by dividing them into up- and downregulated genes based on the fold change direction. Then, we used Wilcoxon sum rank tests to compare –log10(adj-*p* value) calculated by the DESeq2 of the NEGs and ACEGs in the up- and downregulated gene groups in each dataset. We also calculated the nonparametric Cohen’s *d* to measure the effect size. We found that NEGs were more differentially expressed in the downregulated genes in AD (Fig. 2A, *p* value = 8.969e-11, Cohen’ s *d* = 0.2986), down-regulated genes in ASD (Fig. 2B, *p* value = 2.239e-07), up-regulated genes in HD (Fig. 2C, *p* value = 4.772e-9, Cohen’ s *d* = 0.2670), and upregulated genes in SCZ (Fig. 2F, *p* value = 0.001217, Cohen’ s *d* = 0.2269) than ACEGs (Supplementary Table 7). We also ran a permutation test as negative control, which randomly sampled 3,140 and 1,451 genes (same sizes as the NEGs and ACEGs) from the nonessential gene pool as a control distribution. The permutation was conducted 1,000 times, and the *p* values from the two-sided Wilcoxon rank-sum tests are shown in Supplementary Fig. 4.

### NEGs and ACEGs are enriched in different co-expression networks and show different temporal expression patterns

**Fig. 3:**
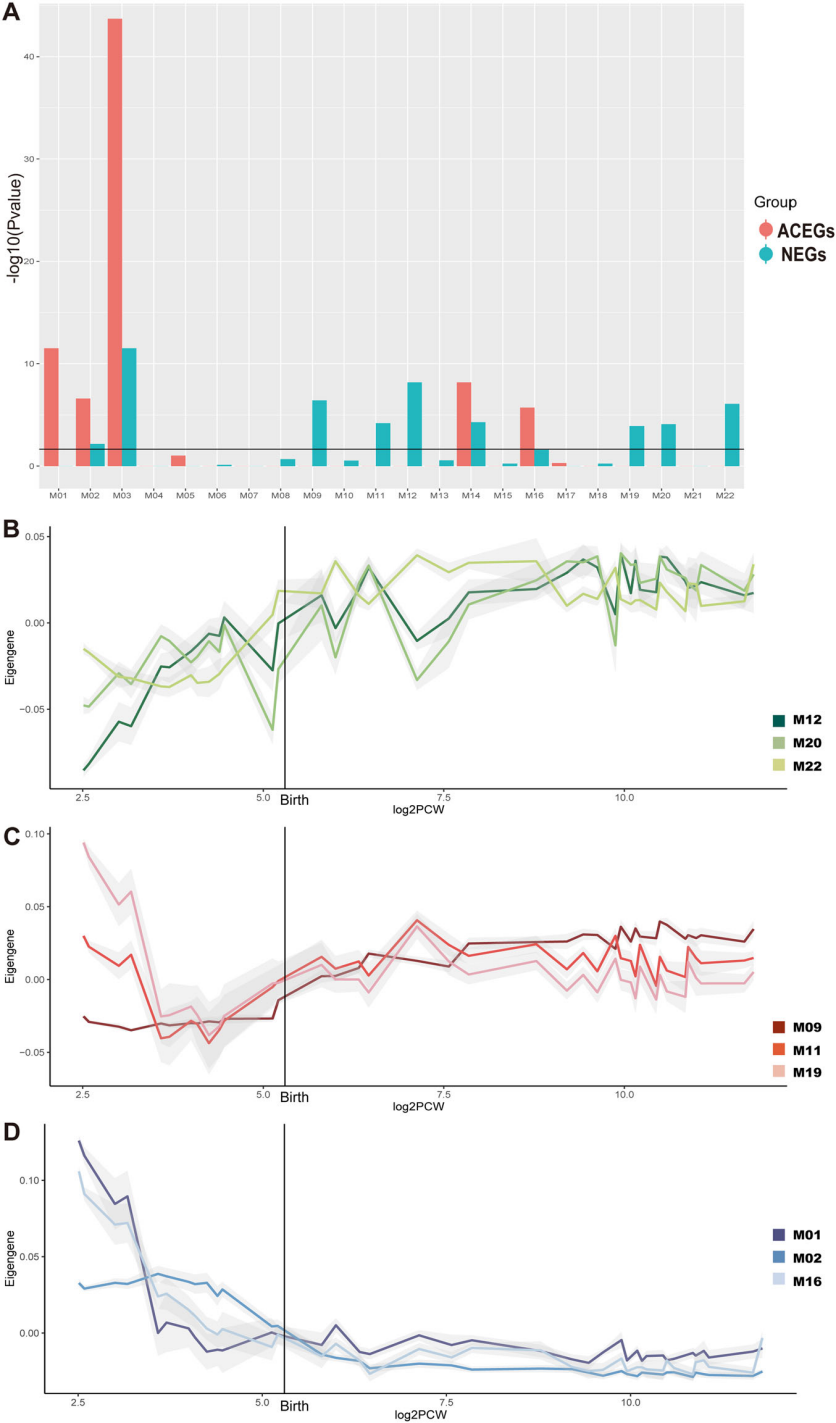
Neuronal essential genes (NEGs) and Achilles Project-specific essential genes (ACEGs) are enriched in different modules and show different expression patterns. **a** Module enrichment of NEGs and ACEGs. **b** Eigengene expression patterns of modules M12, M20, and M22. **c** Eigengene expression patterns of modules M11, M9, and M19. **d** Eigengene expression patterns of modules M01, M02, and M16.

We downloaded the BrainSpan spatio-temporal transcriptome gene-level data of the human brain (GSE25219) from the GEO database and applied WGCNA^34^ to the data. We identified 22 co-expression modules. We found that NEGs were significantly enriched (FDR < 0.05, Fisher’s exact test) in 6 modules (M09, M11, M12, M19, M20, M22), while ACEGs were enriched in three modules (M01, M02, M16) (Fig. 3A and Supplementary Fig. 5). GO analysis showed that NEGs-enriched M12 and M20 molecules are enriched in synapse and neuron projection, while ACEGs-enriched modules M01, M02, and M16 are enriched in basic and general functions such as cell cycle, nucleic acid processing, and chromosomal parts (Supplementary Fig. 6). We then calculated the eigengene expression of these modules and categorized them by expression patterns: M12, M20, M22 showed a graduate increased expression level before birth, then maintained at a relative high level through the old ages (up to 70 years old) (Fig. 3B). M11, M9, M19 exhibited relatively low expression level before birth, but remained at a median expression throughout mid-age (Fig. 3C). M01, M02, M16 showed a high expression at the beginning of conception, but decreased before birth and remained at low levels through mid-age (Fig. 3D). Overall, these results suggest that while ACEGs may have important roles exclusively during early brain development, NEGs may be important for both early development and the maintenance of brain function throughout life.

### Association of NEGs and ACEGs with neuropsychiatric genetic risk

**Fig. 4:**
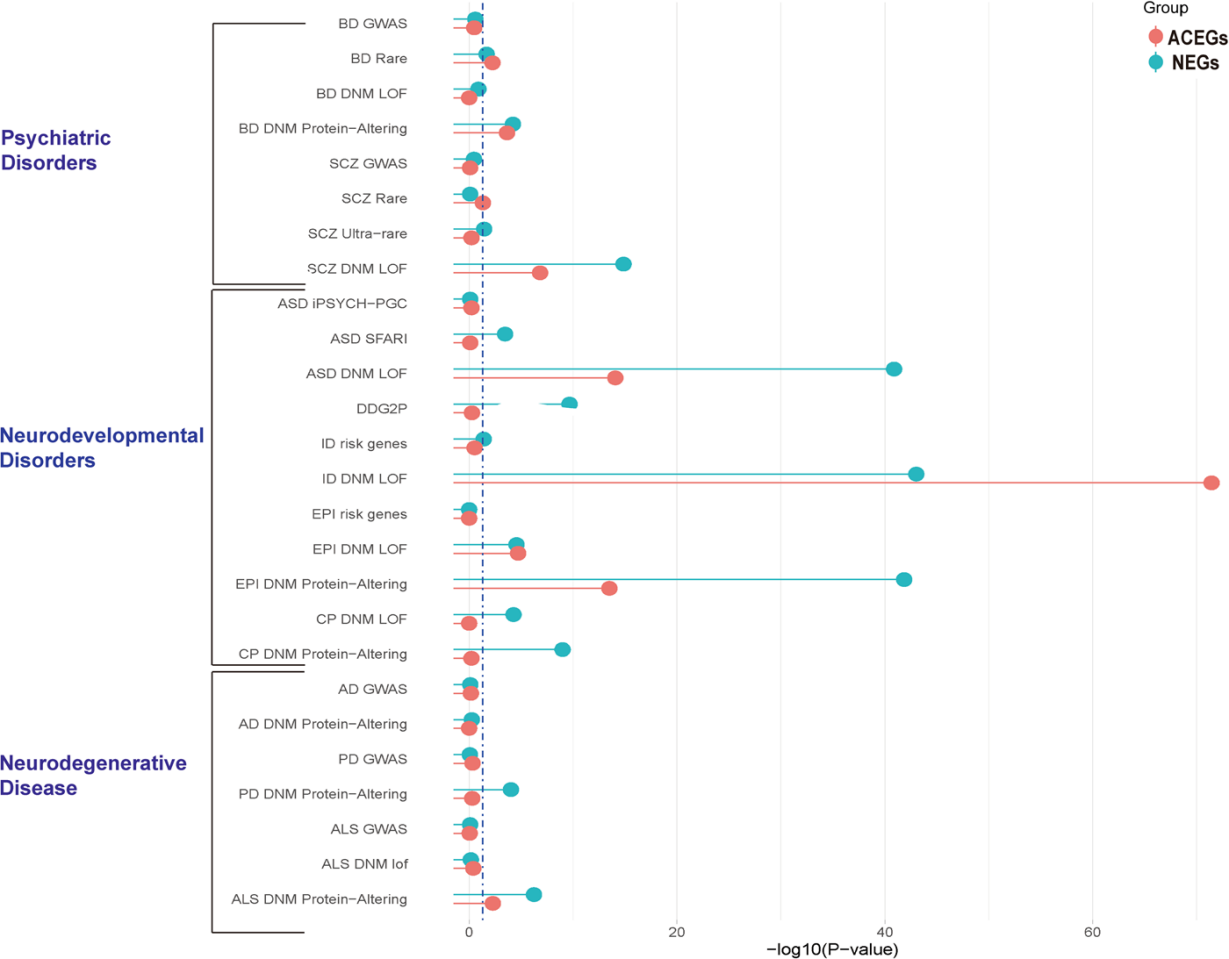
Enrichment of Neuronal Essential Genes (NEGs) and Achilles Project-specific essential genes (ACEGs) in neuropsychiatric disorders’ GWAS, rare variants, and de novo mutations. Enrichment was tested for bipolar disorder (BD), schizophrenia (SCZ), autism spectrum disorder (ASD), developmental disorder (DD), intellectual disability (ID), epilepsy (EPI), cerebral palsy (CP), Alzheimer’s disease (AD), Parkinson’s disease (PD), and amyotrophic lateral sclerosis (ALS).

We obtained GWAS data and rare variants data for neuropsychiatric disorders, as well as de novo mutation data from denovo-db^17^, and then tested the overlap between NEGs and ACEGs among these genetic variants. For GWAS and rare variants data, we used MAGMA^31^ to calculate enrichment p-values; for de novo mutations, we used denovolyzeR^40^, focusing on both LOF (loss-of-function) and protein-altering (missense) mutations. A summary of data and methods used for this analysis is presented in Supplementary Table 8. Both datasets show significant enrichment in BD rare variants (*p* = 0.021 for NEGs, *p* = 5.7 × 10^−3^ for ACEGs) and protein-altering de novo mutations (*p* = 6.1 10^−5^ for NEGs, *p* = 2.3 × 10^−4^ for ACEGs), as well as a strong enrichment in the SCZ loss-of-function de novo mutations (*p* = 1.4 × 10^−15^ for NEGs, *p* = 1.5 × 10^−7^ for ACEGs). NEGs were significantly enriched in the SFARI ASD risk gene list (*p* = 3.5 × 10^−4^), while ACEGs were not (*p* = 0.8). The de novo loss-of-function mutations found in neurodevelopmental disorders patients were enriched in both datasets, such as ASD (*p* = 1.29 × 10^−41^ for NEGs, *p* = 8.65 ×10^−15^ for ACEGs), ID (*p* = 9.37 × 10^−44^ for NEGs, *p* = 3.58 × 10^−72^ for ACEGs), and epilepsy (*p* = 2.77 × 10^−5^ for NEGs, *p* = 1.95 × 10^−5^ for ACEGs). Only NEGs were enriched in protein-altering de novo mutations in PD (*p* = 9.55 × 10^−5^) and ALS (*p* = 5.79 × 10^−7^). These finding demonstrate that ACEGs are exclusively enriched in neurodevelopmental disorders, while NEGs are related to neurodevelopmental, psychiatric, and neurodegenerative diseases (Fig. 4). The *p* values of the enrichment analyses for each module are shown in Supplementary Fig. 7.

### General gene features of NEGs and ACEGs

**Fig. 5:**
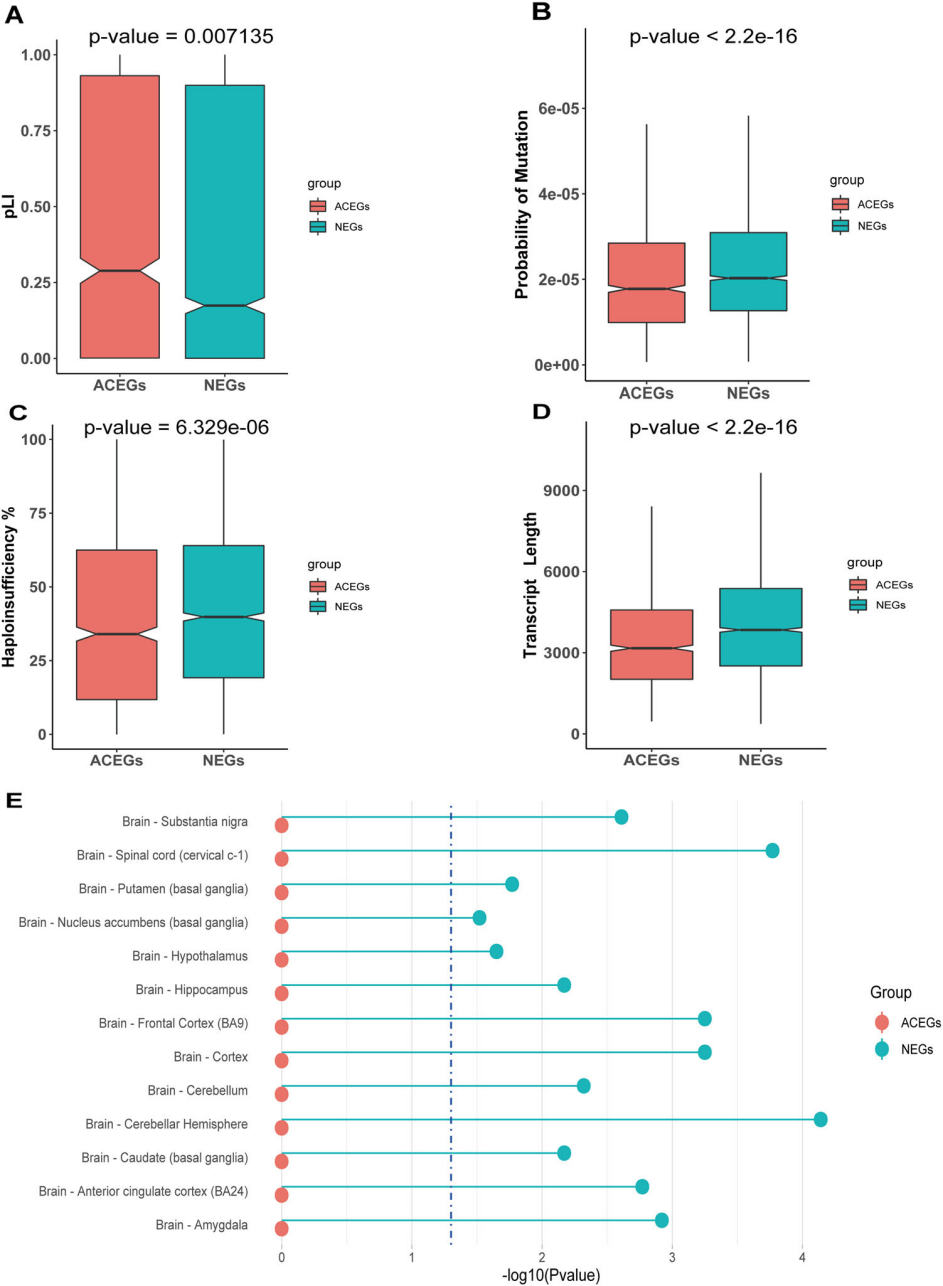
Comparison of probability of loss-of-function intolerance (pLI), probability of mutation, and haploid insufficiency of Neuronal Essential Genes (NEGs) and Achilles Project-specific essential genes (ACEGs). **a** pLI of NEGs and ACEGs. **b** Probability of mutation of NEGs and ACEGs. **c** Haploid insufficiency rate of NEGs and ACEGs. **d** Transcript Length of NEGs and ACEGs. **e** Tissue expression specificities of NEGs and ACEGs.

Finally, we aimed to compare the general features of genes in the two gene sets. The probability of mutation of essential genes was found to be lower than that of unessential genes, while the probability of loss-of-function intolerance (pLI) was higher. Specifically, ACEGs showed significantly lower probability of mutation than NEGs (*p* value < 2.2e-16, Fig. 5B), as well as higher pLI (*p* value = 0.007135, Fig. 5A). Finally, NEGs were more likely to exhibit Haploid insufficiency^41^ (*p* value = 6.329e-6, Fig. 5C). We also used the deTS package^36^ to check the tissue specificities of the two gene sets in GTEx data, with NEGs showing a significant enrichment in several brain regions (Fig. 5E). We used the online tool CSEA^37^ to investigate the expression enrichment of each gene set in particular human brain regions and developmental periods. The results show that NEGs are highly enriched in D2+Spiny neurons in Striatum (Supplementary Fig. 8), but with no obvious enrichment in any specific developmental window (Supplementary Fig. 9). ACEGs are enriched in the early fetal and early mid-fetal period in several brain regions such as amygdala, cortex, hippocampus, and striatum (Supplementary Fig. 10), but not in any specific cell types. (Supplementary Fig. 11). We also compared the GIMS scores of genes in both gene lists, with the results shown in Supplementary Fig. 12.

## Discussion

The majority of in vitro genome-wide screenings of essential genes have been traditionally conducted in cancer cell lines, mainly because of the convenience of cell line studies. After the purification from their usual biological surroundings and the expansion of the specific cell population, researchers can conduct significantly more detailed cellular and molecular analyses in cultured cell lines compared to using a whole organism, in addition to being easier to generate stocks and batches for future reuse of the cells. Because of these advantages, there have been many studies employing cancer cell line screenings to investigate biological processes other than cancer. For example, Lenk et al. used genome-wide screening of essential genes in cancer cell lines to study lysosome function^42^, while Patel et al. used a genome-wide screening approach to study antigen presentation and interferon-γ signaling^11^, both of which are pathways translatable to non-cancer cells.

The mammalian CNS is a very complex system with different types of neurons interacting with glial cells. Accordingly, although applicable to the study of noncancer processes, the traditional in vitro system based on cancer cell line screening may not be able to capture all aspects related to in vivo neurons due to different environment and contexts. Particularly for the study of psychiatric and neurodegenerative diseases, it is highly valuable to conduct such genome-wide screenings in in vivo healthy CNS tissues, obtain “essential gene” lists in cells relevant to the CNS, and then make it publicly available to the scientific community. Moreover, the brain function is thought to be particularly sensitive to the accumulation of deleterious mutations, emphasizing the need for the identification of specific sets of genes in which deleterious mutations affect the survival and function of neurons.

Based on this, and in possession of a recently published list of NEGs derived from mouse brain tissue^14^, we initially identified two gene sets named NEGs and ACEGs for downstream analysis and validation. Not surprisingly, we found that NEGs are enriched in neuronal components and synaptic functions (terms previously reported to be important for mammalian neuron viability^43^), while ACEGs are especially enriched in cell proliferation and general biological processes. These results may help expand the definition of “essential genes” to those genes required not only for survival, but also for the maintenance of cellular functions.

Case-control transcriptome data are widely used tools to detect differentially expressed genes between patients and healthy controls. By applying our lists of essential genes, we found that NEGs are more likely to be differentially expressed in AD, ASD, HD, ALS, and SCZ by comparing the *p* values of DE calculated by DESeq2^27^ of all genes in the different datasets. This may be a result of relatively low expression levels of ACEGs in adult brain tissues. We also used WGCNA^34^ to investigate if the two gene sets belong to distinct co-expression gene networks during brain development using the BrainSpan^35^ transcriptome data. We identified 22 modules in total, three of which were significantly enriched in ACEGs. The eigengene expressions for these ACE-related modules were found to be very high during the early stage of brain development but dropped immediately after birth, indicating that ACEGs have important roles exclusively in the early development. This was confirmed by the CSEA tool^37^. This might be partly because of the common features between neural stem cells and glioma stem cells, which is a component of glioblastomas^44^. On the contrary, the modules enriched in NEGs were found to maintain relatively high expression levels after birth, indicating their important roles in adult neuronal functions.

Genetic data like common variants measured by GWAS^45^, rare variants, and de novo mutations^46^ can identify risk genes for complex multifactorial diseases. We found that both NEGs and ACEGs significantly overlap with the NDD and psychiatric disorders risk genes^47^, such as ASD, ID, EPI, cerebral palsy (CP), and SCZ, while only NEGs are enriched in neurodegenerative disorders (PD and ALS) in de novo mutations. This is consistent with their high expression level in post-development adult brains. Of note, the correlations of disease risk and expression during development were consistent with those reported by Li et al.^48^, which showed that ASD and SCZ risk genes are highly expressed in fetal and infant stages (consistent with our findings that both NEGs and ACEGs are enriched in these two disease risk genes), while PD risk genes are highly expressed in adult (consistent with only NEGs being enriched in PD).

We also investigated some general features of the essential genes from both gene sets. The NEGs showed generally fewer essentiality features than the ACEGs. Specifically, NEGs showed higher probability of mutations and lower probability of loss-of-function intolerance, possibly because NEGs are highly enriched in D2+ spiny neurons in striatum and reflect the essentiality for this specific type of neuron, but not all the cells and the whole organ. NEGs also have longer transcript lengths and higher Haploinsufficiency scores, which are consistent with reports that genes with higher Haploinsufficiency score tend to have longer transcripts and greater tissue specificity^41^. The results using deTS R package and GTEx data as a reference showed that NEGs have more specific expression in brain tissue. Of note, the reference data of brain regions and cell types used in this analysis come from bulk tissue RNA-Seq, i.e., they may not be precise enough when comparing to reference data from single-cell sequencing technologies.

Both NEGs and ACEGs gene lists have their own limitations. The in vitro cancer cell line genome-wide screenings are much easier to conduct, and we found that the ACEGs derived from them surprisingly captured the highly expressed and necessary genes in the early stages of brain development. Meanwhile, NEGs may be particularly influenced by a potential species bias, given significant differences between the human and mouse brain. The potential use of cerebral organoids from human induced pluripotent stem cells may be a better choice for future CNS screenings. Nevertheless, before cerebral organoids can confidently recapitulate features of human brain, NEGs obtained from such in vivo CNS screenings in adult mice may still be a good source of information for the study of neurodegenerative and neuropsychiatric diseases. Of note, given that NEGs represent genes required for the maintenance of adult neurons that strongly overlap with known risk genes for neurodegenerative diseases, they may represent a valuable reference gene set for the study of these conditions. For example, a higher risk for neurodegenerative disorders may be identified in individuals that carry a number of deleterious genetic variants or present with low mRNA levels of genes belonging to the NEGs list. In contrast, the ACEGs captured the common essentiality of many immortalized cancer cell lines of different tissue origins. Cancers commonly result from somatic mutations disrupting normal controls of the cell cycle, which renders their classification as developmental diseases. That may be part of the reason why ACEGs significantly overlapped with risk genes in several NDDs.

In this study, we put two essential gene sets obtained from distinct cell-based genome-wide screenings of essential genes into the neuropsychiatric context. Both gene sets significantly overlapped with neurodevelopmental, neurodegenerative, and psychiatric disorders risk genes, although only NEGs showed enrichment in the neurodegenerative disorders. Overall, our data suggest that essential genes from cell-based screening, especially in vivo CNS screenings of healthy tissue, can provide insights into potential risk genes and pathogenic variants underlying human brain disorders. Future genome-wide in vivo screenings in different mouse models and cerebral organoids from patients with neuropsychiatric and neurodegenerative diseases hold the promise to shed light on important aspects of these conditions.

## Supplementary information

Supplementary Material

## Data Availability

The codes used to generate results of this study are available from the corresponding author upon request.
