# Supplementary material for: Essential genes from genome-wide screenings as a resource for neuropsychiatric disorders gene discovery
Source: Transl Psychiatry. 2021 May 25;11:317. doi: 10.1038/s41398-021-01447-y (PMC8149887; doi:10.1038/s41398-021-01447-y)
Supplement: Supplementary file 1 — Supplementary Material [file 41398_2021_1447_MOESM1_ESM.docx]

**Supplementary Data**

**
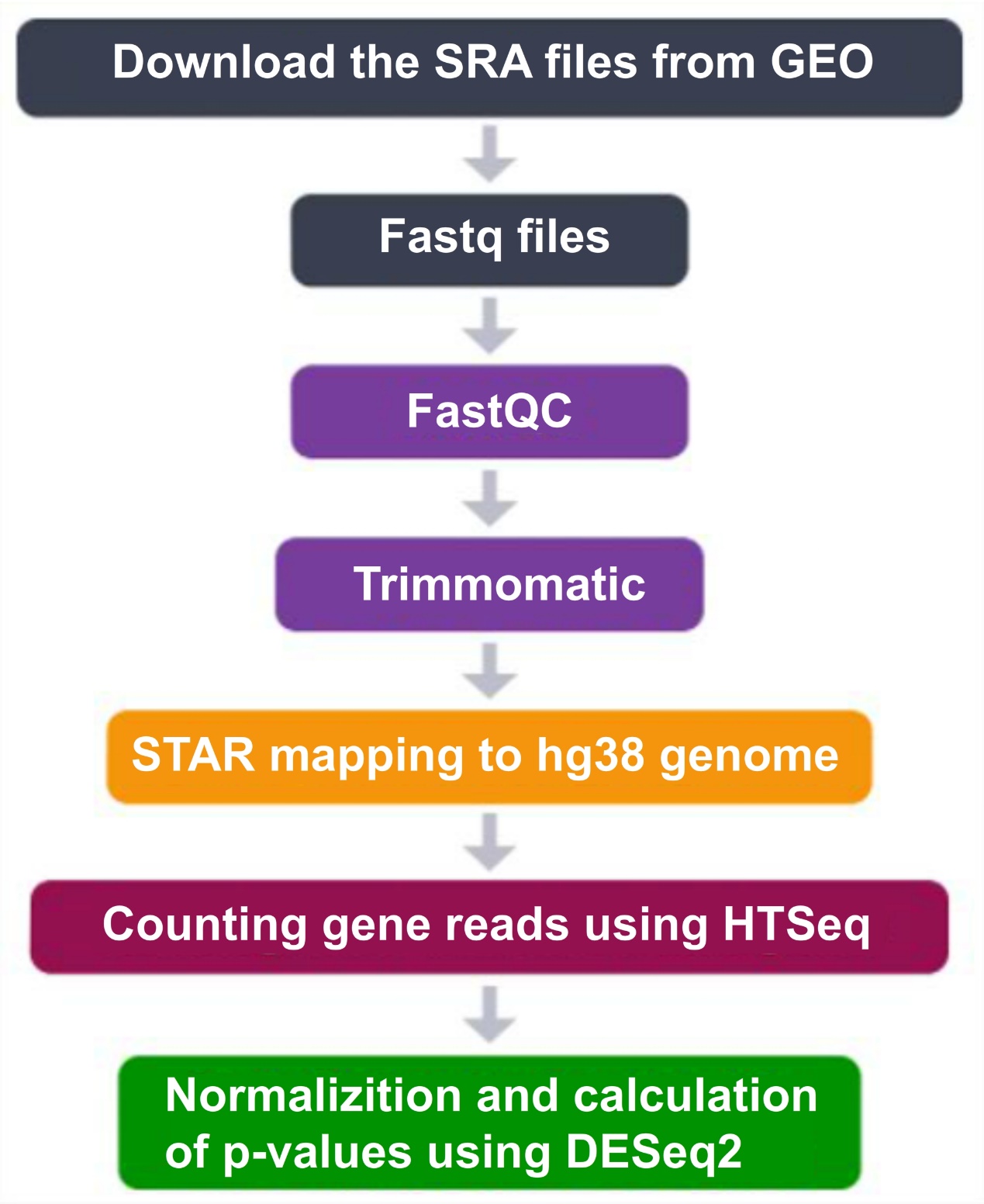
**

**Supplementary Figure 1.** Schematic representation of the RNA-Seq analysis workflow.

**
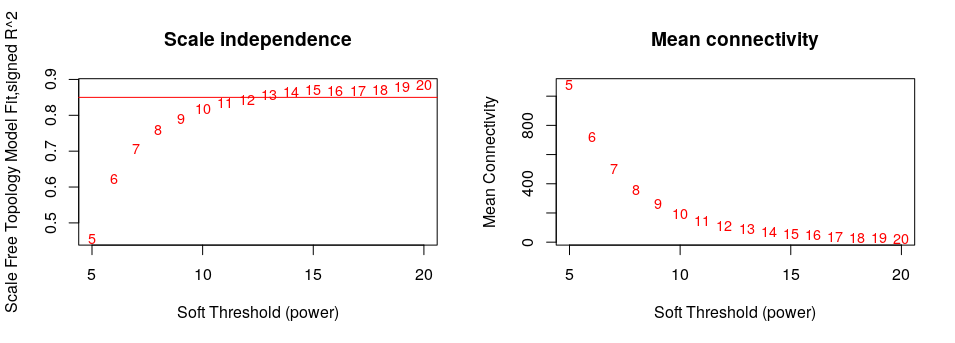
**

**Supplementary Figure 2.** Soft power picking to archive scale free topology model for the BrainSpan data.

**
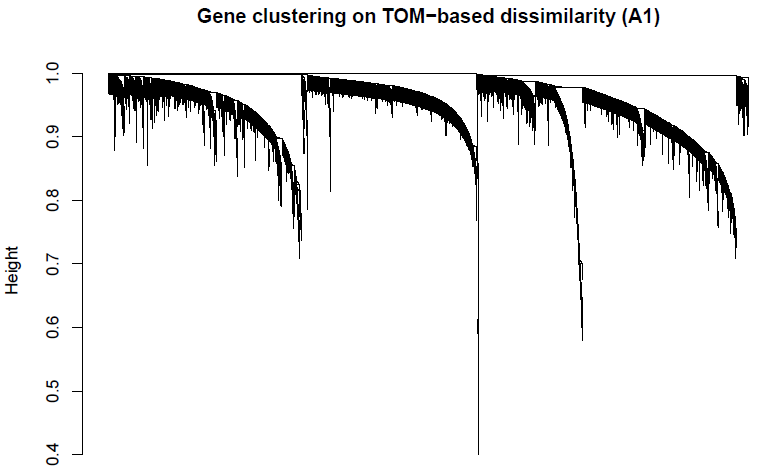
**

**Supplementary Figure 3.** The dendrogram based on co-expression topological model.

**
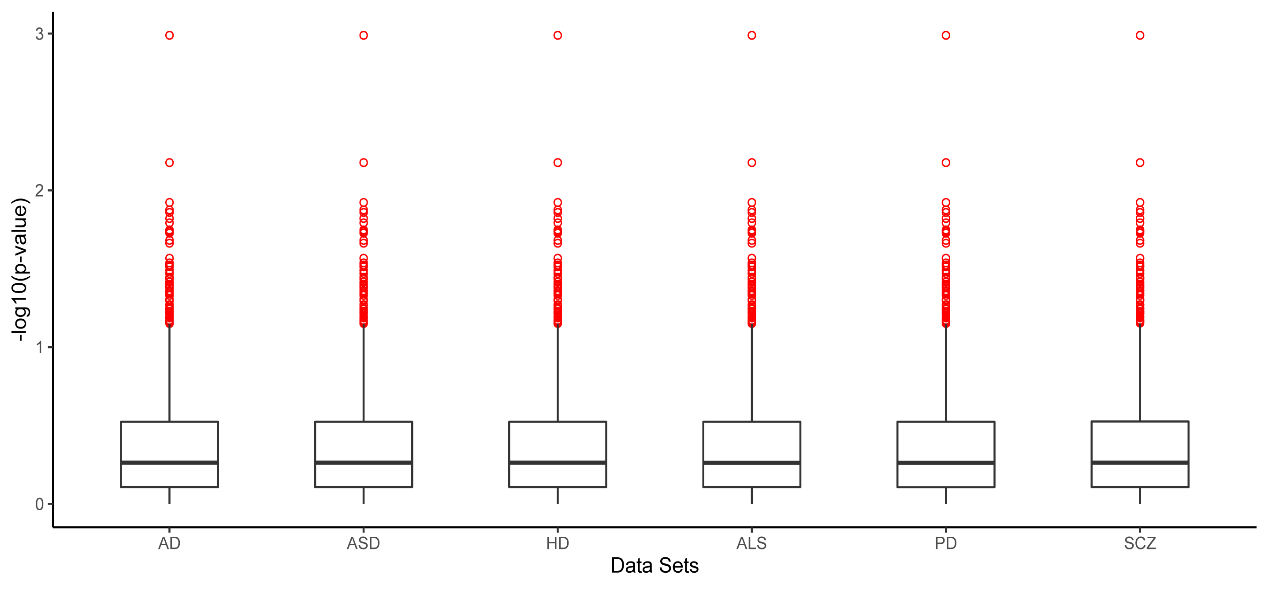
**

**Supplementary Figure 4.** The -log10(p-value) of the two-tailed Wilcox test results from 1000 times permutation test.

**
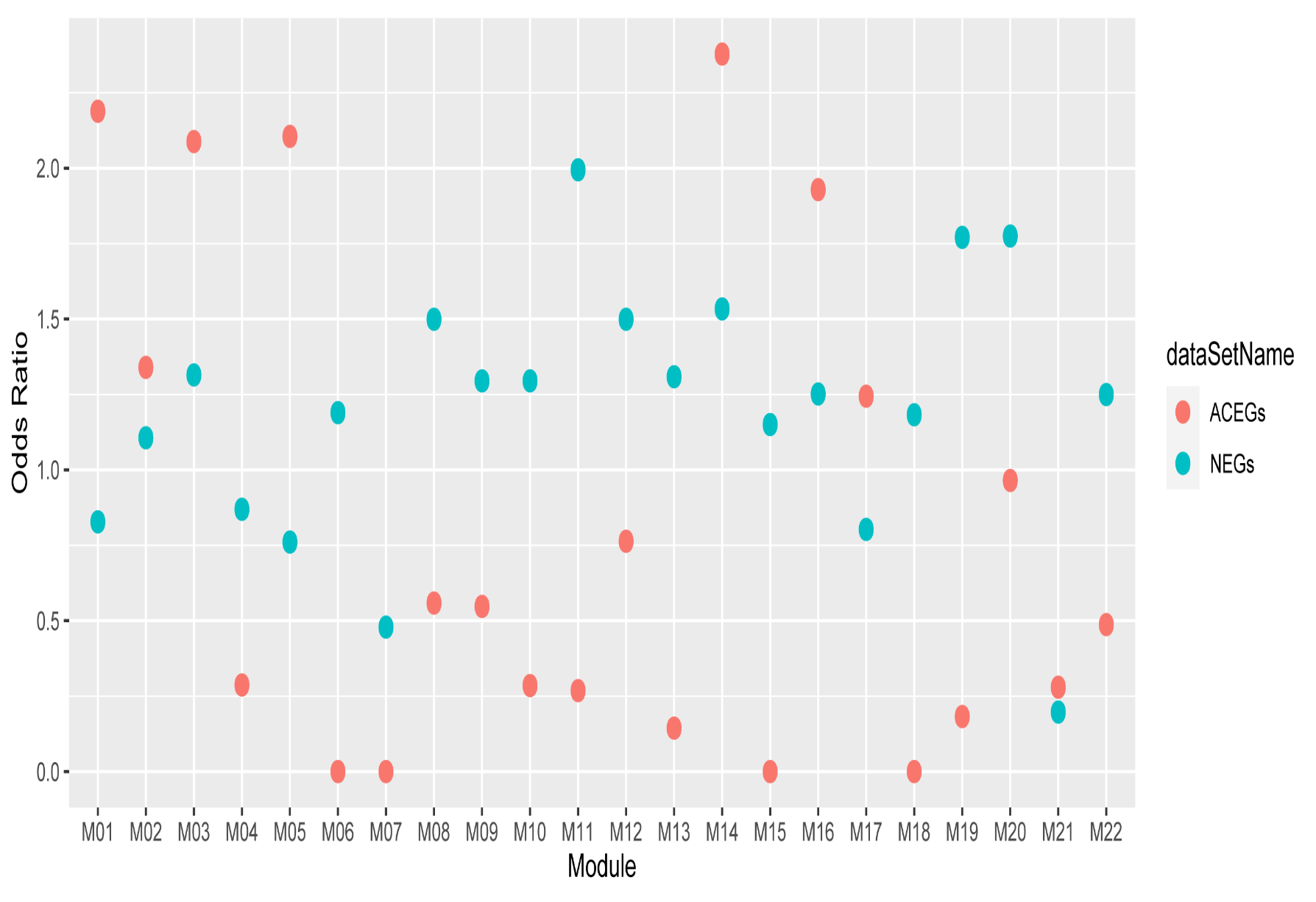
**

**Supplementary Figure 5.** Odds ratio of Fisher's test for whether NEGs or ACEGs were significantly enriched in modules.

**
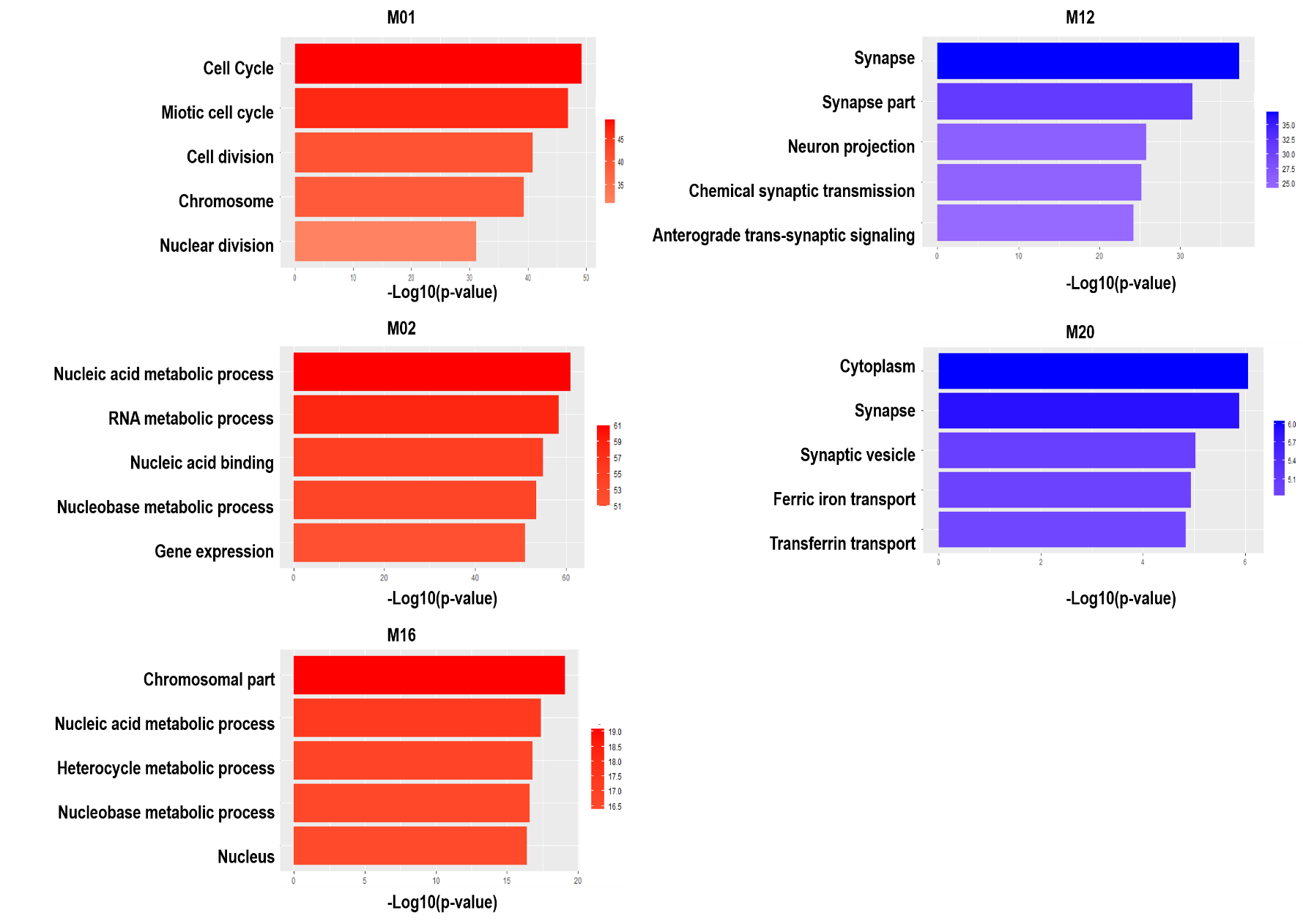
**

**Supplementary Figure 6.** Top GO terms of Module 01, Module 02, Module 16, Module 12, Module 20.

**
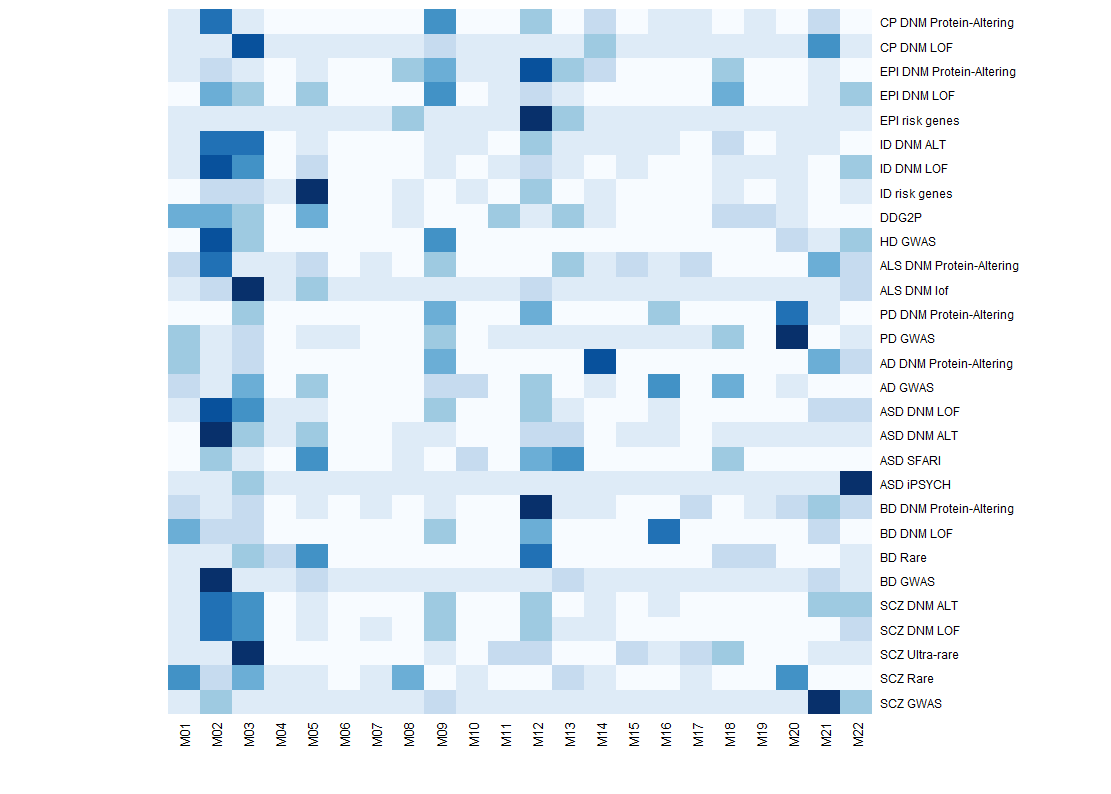
**

**Supplementary Figure 7.** Enrichment of each module with neuropsychiatric risk genes.

**
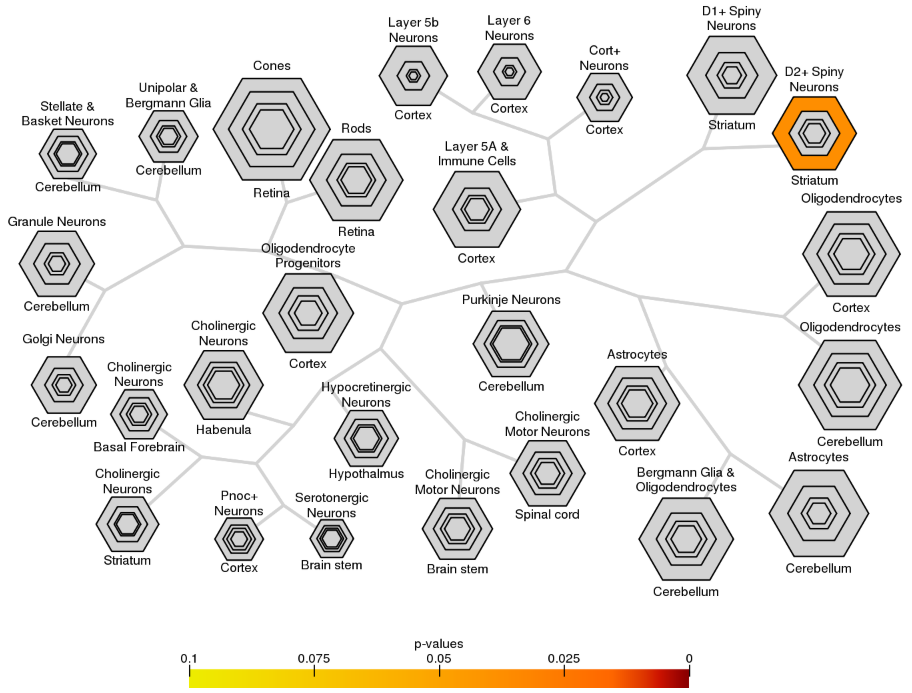
**

**Supplementary Figure 8.** Enrichment of NEGs in D2+Spiny Neurons in Striatum.

**
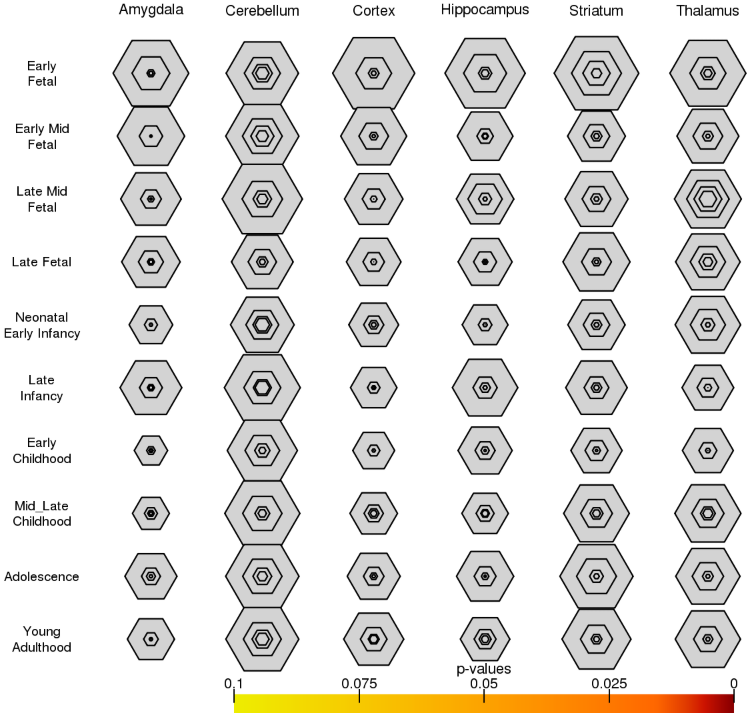
**

**Supplementary Figure 9.** Enrichment of the NEGs in the developmental stage and brain regions.

**
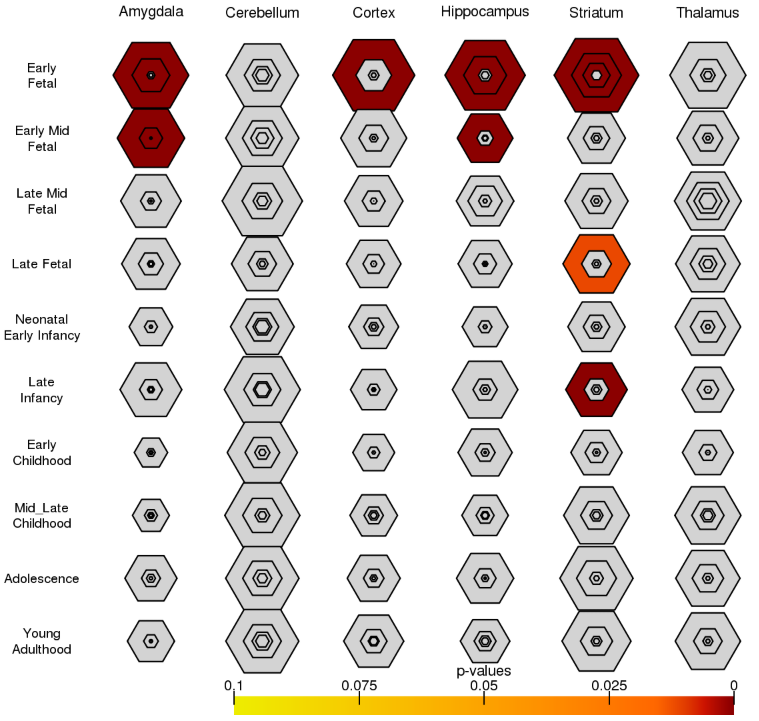
**

**Supplementary Figure 10.** Enrichment of the ACEGs in the developmental stage and brain regions.

**
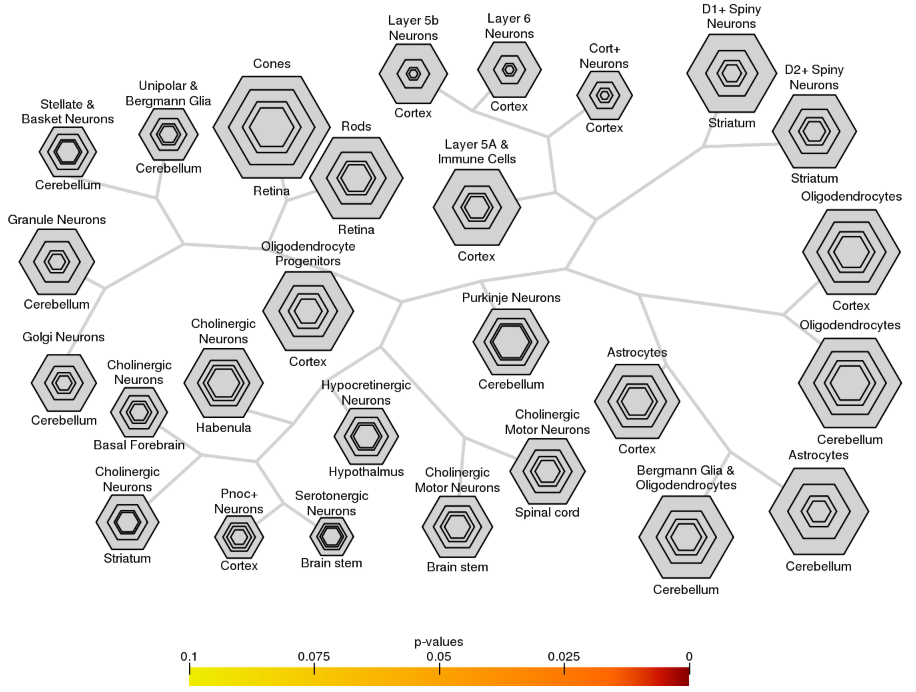
**

**Supplementary Figure 11.** Enrichment of ACEGs in different cell types.


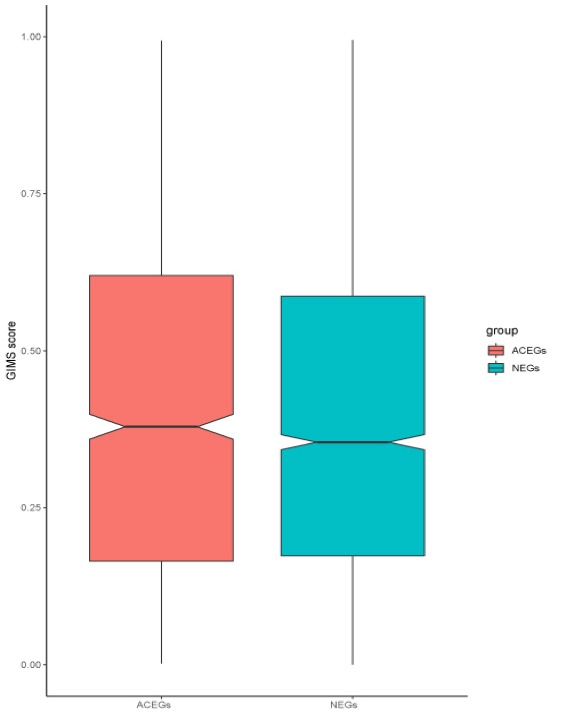


p-value = 0.3803

**Supplementary Figure 12.** Wilcoxon rank sum test of the Gene-level Integrated Metric of negative Selection (GIMS) scores of two gene lists.

**Supplementary Table 1.** Enriched GO terms (Biological Processing) with false discovery date < 0.05 for Neuronal Essential Genes (NEGs).

| **Gene Set** | **Description** | **Size** | **Expect** | **Ratio** | **P Value** | **FDR** |
| --- | --- | --- | --- | --- | --- | --- |
| GO:0046777 | protein autophosphorylation | 225 | 38.065 | 2.4432 | 0 | 0 |
| GO:0060627 | regulation of vesicle-mediated transport | 499 | 84.419 | 1.7887 | 5.04E-14 | 2.14E-11 |
| GO:0099003 | vesicle-mediated transport in synapse | 203 | 34.343 | 2.2712 | 1.58E-13 | 4.46E-11 |
| GO:0007265 | Ras protein signal transduction | 437 | 73.93 | 1.826 | 2.10E-13 | 4.46E-11 |
| GO:0061919 | process utilizing autophagic mechanism | 473 | 80.021 | 1.7495 | 2.73E-12 | 4.64E-10 |
| GO:0099504 | synaptic vesicle cycle | 193 | 32.651 | 2.2051 | 7.17E-12 | 9.94E-10 |
| GO:0010975 | regulation of neuron projection development | 475 | 80.359 | 1.7297 | 8.19E-12 | 9.94E-10 |
| GO:0061564 | axon development | 490 | 82.897 | 1.7009 | 2.21E-11 | 2.35E-09 |
| GO:0097191 | extrinsic apoptotic signaling pathway | 220 | 37.219 | 2.0688 | 5.20E-11 | 4.91E-09 |
| GO:0051648 | vesicle localization | 305 | 51.599 | 1.8799 | 9.05E-11 | 7.69E-09 |
| GO:0071900 | regulation of protein serine/threonine kinase activity | 497 | 84.081 | 1.6651 | 1.35E-10 | 1.04E-08 |
| GO:1990778 | protein localization to cell periphery | 301 | 50.922 | 1.8656 | 2.25E-10 | 1.59E-08 |
| GO:0048284 | organelle fusion | 121 | 20.47 | 2.3937 | 6.23E-10 | 4.07E-08 |
| GO:0018209 | peptidyl-serine modification | 305 | 51.599 | 1.783 | 5.63E-09 | 3.42E-07 |
| GO:0006979 | response to oxidative stress | 431 | 72.915 | 1.6457 | 6.10E-09 | 3.46E-07 |
| GO:0031346 | positive regulation of cell projection organization | 365 | 61.75 | 1.7004 | 8.40E-09 | 4.46E-07 |
| GO:0016050 | vesicle organization | 313 | 52.952 | 1.7563 | 1.07E-08 | 5.34E-07 |
| GO:0006790 | sulfur compound metabolic process | 354 | 59.889 | 1.7032 | 1.25E-08 | 5.91E-07 |
| GO:0050769 | positive regulation of neurogenesis | 447 | 75.622 | 1.6133 | 1.62E-08 | 7.25E-07 |
| GO:1902532 | negative regulation of intracellular signal transduction | 495 | 83.742 | 1.5643 | 3.60E-08 | 1.53E-06 |
| GO:0017156 | calcium ion regulated exocytosis | 152 | 25.715 | 2.0611 | 5.91E-08 | 2.39E-06 |
| GO:2001233 | regulation of apoptotic signaling pathway | 385 | 65.133 | 1.6274 | 8.77E-08 | 3.39E-06 |
| GO:0042326 | negative regulation of phosphorylation | 423 | 71.562 | 1.593 | 9.97E-08 | 3.68E-06 |
| GO:0031098 | stress-activated protein kinase signaling cascade | 305 | 51.599 | 1.7055 | 1.16E-07 | 3.96E-06 |
| GO:0048638 | regulation of developmental growth | 314 | 53.121 | 1.6942 | 1.16E-07 | 3.96E-06 |
| GO:0048193 | Golgi vesicle transport | 366 | 61.919 | 1.6312 | 1.56E-07 | 4.96E-06 |
| GO:0061025 | membrane fusion | 148 | 25.038 | 2.0369 | 1.60E-07 | 4.96E-06 |
| GO:0031647 | regulation of protein stability | 267 | 45.17 | 1.7489 | 1.63E-07 | 4.96E-06 |
| GO:0048017 | inositol lipid-mediated signaling | 165 | 27.914 | 1.9703 | 1.88E-07 | 5.52E-06 |
| GO:0097485 | neuron projection guidance | 260 | 43.986 | 1.7506 | 2.24E-07 | 6.35E-06 |
| GO:0140029 | exocytic process | 84 | 14.211 | 2.3925 | 2.58E-07 | 7.05E-06 |
| GO:0022604 | regulation of cell morphogenesis | 473 | 80.021 | 1.5371 | 2.65E-07 | 7.05E-06 |
| GO:0070371 | ERK1 and ERK2 cascade | 326 | 55.152 | 1.65 | 3.67E-07 | 9.45E-06 |
| GO:0070997 | neuron death | 325 | 54.982 | 1.6369 | 6.21E-07 | 1.55E-05 |
| GO:1901652 | response to peptide | 487 | 82.389 | 1.5051 | 7.94E-07 | 1.92E-05 |
| GO:0016358 | dendrite development | 219 | 37.05 | 1.7814 | 8.12E-07 | 1.92E-05 |
| GO:0009259 | ribonucleotide metabolic process | 500 | 84.588 | 1.4896 | 1.16E-06 | 2.67E-05 |
| GO:0098876 | vesicle-mediated transport to the plasma membrane | 93 | 15.733 | 2.2246 | 1.35E-06 | 3.03E-05 |
| GO:0023061 | signal release | 456 | 77.145 | 1.5037 | 1.88E-06 | 4.11E-05 |
| GO:0035329 | hippo signaling | 38 | 6.4287 | 2.9555 | 2.71E-06 | 5.76E-05 |
| GO:0099177 | regulation of trans-synaptic signaling | 417 | 70.547 | 1.5167 | 3.08E-06 | 6.38E-05 |
| GO:0046486 | glycerolipid metabolic process | 456 | 77.145 | 1.4907 | 3.26E-06 | 6.59E-05 |
| GO:0043087 | regulation of GTPase activity | 472 | 79.851 | 1.4777 | 3.83E-06 | 7.57E-05 |
| GO:0001667 | ameboidal-type cell migration | 381 | 64.456 | 1.5359 | 3.99E-06 | 7.71E-05 |
| GO:0016049 | cell growth | 473 | 80.021 | 1.4746 | 4.27E-06 | 8.06E-05 |
| GO:0010038 | response to metal ion | 356 | 60.227 | 1.5442 | 6.13E-06 | 0.000113 |
| GO:0007249 | I-kappaB kinase/NF-kappaB signaling | 259 | 43.817 | 1.6432 | 6.92E-06 | 0.000123 |
| GO:0010256 | endomembrane system organization | 400 | 67.671 | 1.5073 | 6.96E-06 | 0.000123 |
| GO:0035418 | protein localization to synapse | 61 | 10.32 | 2.4225 | 7.47E-06 | 0.00013 |
| GO:0051656 | establishment of organelle localization | 494 | 83.573 | 1.4478 | 8.32E-06 | 0.000141 |
| GO:0032147 | activation of protein kinase activity | 321 | 54.306 | 1.5652 | 8.70E-06 | 0.000143 |
| GO:0006644 | phospholipid metabolic process | 470 | 79.513 | 1.4589 | 8.78E-06 | 0.000143 |
| GO:0060560 | developmental growth involved in morphogenesis | 225 | 38.065 | 1.6813 | 9.90E-06 | 0.000159 |
| GO:0045927 | positive regulation of growth | 250 | 42.294 | 1.6314 | 1.38E-05 | 0.000218 |
| GO:0009743 | response to carbohydrate | 225 | 38.065 | 1.6551 | 1.99E-05 | 0.000307 |
| GO:0048008 | platelet-derived growth factor receptor signaling pathway | 53 | 8.9664 | 2.4536 | 2.05E-05 | 0.000311 |
| GO:0022406 | membrane docking | 177 | 29.944 | 1.7366 | 2.51E-05 | 0.000374 |
| GO:0035690 | cellular response to drug | 349 | 59.043 | 1.5074 | 2.62E-05 | 0.000384 |
| GO:0006732 | coenzyme metabolic process | 354 | 59.889 | 1.5028 | 2.68E-05 | 0.000386 |
| GO:0018212 | peptidyl-tyrosine modification | 389 | 65.81 | 1.4739 | 3.04E-05 | 0.000431 |
| GO:0048732 | gland development | 434 | 73.423 | 1.4437 | 3.38E-05 | 0.000471 |
| GO:0051348 | negative regulation of transferase activity | 266 | 45.001 | 1.5777 | 3.51E-05 | 0.000475 |
| GO:0018210 | peptidyl-threonine modification | 114 | 19.286 | 1.9185 | 3.52E-05 | 0.000475 |
| GO:0016197 | endosomal transport | 212 | 35.865 | 1.645 | 4.35E-05 | 0.000578 |
| GO:0046677 | response to antibiotic | 316 | 53.46 | 1.5152 | 4.89E-05 | 0.00064 |
| GO:0051668 | localization within membrane | 146 | 24.7 | 1.7814 | 5.25E-05 | 0.000676 |
| GO:0048511 | rhythmic process | 279 | 47.2 | 1.5466 | 5.58E-05 | 0.000707 |
| GO:1903008 | organelle disassembly | 96 | 16.241 | 1.9703 | 6.51E-05 | 0.000813 |
| GO:0045862 | positive regulation of proteolysis | 343 | 58.028 | 1.4821 | 6.83E-05 | 0.00083 |
| GO:0016311 | dephosphorylation | 461 | 77.99 | 1.4104 | 6.84E-05 | 0.00083 |
| GO:0048013 | ephrin receptor signaling pathway | 80 | 13.534 | 2.0688 | 6.94E-05 | 0.00083 |
| GO:0009896 | positive regulation of catabolic process | 409 | 69.193 | 1.4308 | 8.75E-05 | 0.001034 |
| GO:0051056 | regulation of small GTPase mediated signal transduction | 331 | 55.998 | 1.4822 | 9.03E-05 | 0.001045 |
| GO:0010821 | regulation of mitochondrion organization | 176 | 29.775 | 1.6793 | 9.19E-05 | 0.001045 |
| GO:0033865 | nucleoside bisphosphate metabolic process | 136 | 23.008 | 1.782 | 9.22E-05 | 0.001045 |
| GO:0001101 | response to acid chemical | 332 | 56.167 | 1.4777 | 0.000101 | 0.001128 |
| GO:1902115 | regulation of organelle assembly | 209 | 35.358 | 1.6121 | 0.000106 | 0.001176 |
| GO:1901293 | nucleoside phosphate biosynthetic process | 343 | 58.028 | 1.4648 | 0.000116 | 0.001261 |
| GO:0008637 | apoptotic mitochondrial changes | 116 | 19.625 | 1.8344 | 0.000126 | 0.001359 |
| GO:0051051 | negative regulation of transport | 458 | 77.483 | 1.3939 | 0.00013 | 0.001368 |
| GO:0009314 | response to radiation | 443 | 74.945 | 1.401 | 0.000131 | 0.001368 |
| GO:0072522 | purine-containing compound biosynthetic process | 267 | 45.17 | 1.5276 | 0.000132 | 0.001368 |
| GO:0198738 | cell-cell signaling by wnt | 459 | 77.652 | 1.3908 | 0.000143 | 0.001448 |
| GO:0010498 | proteasomal protein catabolic process | 449 | 75.96 | 1.3955 | 0.000143 | 0.001448 |
| GO:0006836 | neurotransmitter transport | 268 | 45.339 | 1.5219 | 0.000149 | 0.001459 |
| GO:0042176 | regulation of protein catabolic process | 370 | 62.595 | 1.4378 | 0.00015 | 0.001459 |
| GO:0001655 | urogenital system development | 326 | 55.152 | 1.4687 | 0.000151 | 0.001459 |
| GO:0050817 | coagulation | 326 | 55.152 | 1.4687 | 0.000151 | 0.001459 |
| GO:0009132 | nucleoside diphosphate metabolic process | 117 | 19.794 | 1.8188 | 0.000153 | 0.001462 |
| GO:0016482 | cytosolic transport | 157 | 26.561 | 1.6942 | 0.000161 | 0.001521 |
| GO:0031532 | actin cytoskeleton reorganization | 96 | 16.241 | 1.9088 | 0.000163 | 0.001523 |
| GO:0007163 | establishment or maintenance of cell polarity | 203 | 34.343 | 1.6015 | 0.000168 | 0.001552 |
| GO:0006520 | cellular amino acid metabolic process | 318 | 53.798 | 1.4685 | 0.000182 | 0.001664 |
| GO:0071241 | cellular response to inorganic substance | 209 | 35.358 | 1.5838 | 0.000202 | 0.001825 |
| GO:0032409 | regulation of transporter activity | 247 | 41.787 | 1.5316 | 0.000209 | 0.001874 |
| GO:0006909 | phagocytosis | 238 | 40.264 | 1.5398 | 0.000223 | 0.001972 |
| GO:1990089 | response to nerve growth factor | 53 | 8.9664 | 2.2306 | 0.000232 | 0.002034 |
| GO:0009895 | negative regulation of catabolic process | 282 | 47.708 | 1.4882 | 0.000248 | 0.002149 |
| GO:0097327 | response to antineoplastic agent | 94 | 15.903 | 1.8865 | 0.00026 | 0.002233 |
| GO:0007205 | protein kinase C-activating G protein-coupled receptor signaling pathway | 28 | 4.7369 | 2.7444 | 0.00027 | 0.002291 |
| GO:0021915 | neural tube development | 161 | 27.237 | 1.6521 | 0.0003 | 0.002407 |
| GO:0046390 | ribose phosphate biosynthetic process | 255 | 43.14 | 1.5067 | 0.000308 | 0.002445 |
| GO:0007033 | vacuole organization | 157 | 26.561 | 1.6566 | 0.000327 | 0.002508 |
| GO:0009141 | nucleoside triphosphate metabolic process | 304 | 51.43 | 1.4583 | 0.000328 | 0.002508 |
| GO:1905475 | regulation of protein localization to membrane | 177 | 29.944 | 1.603 | 0.000413 | 0.003026 |
| GO:1901654 | response to ketone | 189 | 31.974 | 1.5638 | 0.000591 | 0.004152 |
| GO:0099072 | regulation of postsynaptic membrane neurotransmitter receptor levels | 65 | 10.996 | 2.0006 | 0.000682 | 0.00472 |
| GO:1901342 | regulation of vasculature development | 313 | 52.952 | 1.4164 | 0.000812 | 0.005569 |
| GO:0002446 | neutrophil mediated immunity | 496 | 83.912 | 1.3228 | 0.000857 | 0.005738 |
| GO:0009410 | response to xenobiotic stimulus | 282 | 47.708 | 1.4253 | 0.001163 | 0.007271 |
| GO:0051961 | negative regulation of nervous system development | 297 | 50.245 | 1.4131 | 0.001178 | 0.007308 |
| GO:0009914 | hormone transport | 322 | 54.475 | 1.3768 | 0.001861 | 0.010722 |
| GO:0051271 | negative regulation of cellular component movement | 301 | 50.922 | 1.3746 | 0.002692 | 0.01448 |
| GO:0032970 | regulation of actin filament-based process | 362 | 61.242 | 1.3226 | 0.004027 | 0.020496 |
| GO:0046683 | response to organophosphorus | 138 | 23.346 | 1.542 | 0.004141 | 0.02095 |
| GO:0060759 | regulation of response to cytokine stimulus | 163 | 27.576 | 1.4868 | 0.004698 | 0.023488 |
| GO:0010959 | regulation of metal ion transport | 360 | 60.904 | 1.2971 | 0.007437 | 0.033987 |
| GO:0002764 | immune response-regulating signaling pathway | 485 | 82.051 | 1.2431 | 0.009605 | 0.040418 |
| GO:0044262 | cellular carbohydrate metabolic process | 271 | 45.847 | 1.3305 | 0.010032 | 0.041565 |
| GO:0043648 | dicarboxylic acid metabolic process | 107 | 18.102 | 1.5468 | 0.010115 | 0.041565 |

**Supplementary Table 2.** Enriched GO terms (Cellular Component) with false discovery date < 0.05 for Neuronal Essential Genes (NEGs).

| **Gene Set** | **Description** | **Size** | **Expect** | **Ratio** | **P Value** | **FDR** |
| --- | --- | --- | --- | --- | --- | --- |
| GO:0098793 | presynapse | 483 | 86.794 | 1.613 | 7.93E-10 | 1.31E-07 |
| GO:0010008 | endosome membrane | 461 | 82.841 | 1.6176 | 1.53E-09 | 1.31E-07 |
| GO:0030139 | endocytic vesicle | 289 | 51.933 | 1.733 | 2.50E-08 | 1.43E-06 |
| GO:0031300 | intrinsic component of organelle membrane | 227 | 40.792 | 1.6915 | 2.81E-06 | 0.000105 |
| GO:0098978 | glutamatergic synapse | 350 | 62.894 | 1.5423 | 3.13E-06 | 0.000105 |
| GO:0033267 | axon part | 374 | 67.207 | 1.5177 | 3.82E-06 | 0.000105 |
| GO:0031252 | cell leading edge | 393 | 70.621 | 1.501 | 4.27E-06 | 0.000105 |
| GO:0005769 | early endosome | 339 | 60.918 | 1.5266 | 7.87E-06 | 0.000169 |
| GO:0030135 | coated vesicle | 275 | 49.417 | 1.5784 | 1.17E-05 | 0.000223 |
| GO:0098685 | Schaffer collateral - CA1 synapse | 82 | 14.735 | 2.1038 | 1.65E-05 | 0.000284 |
| GO:0031201 | SNARE complex | 47 | 8.4458 | 2.4864 | 2.05E-05 | 0.000295 |
| GO:0005774 | vacuolar membrane | 397 | 71.34 | 1.4578 | 2.06E-05 | 0.000295 |
| GO:0030133 | transport vesicle | 374 | 67.207 | 1.4582 | 3.51E-05 | 0.000465 |
| GO:0048770 | pigment granule | 105 | 18.868 | 1.908 | 4.32E-05 | 0.000531 |
| GO:0019898 | extrinsic component of membrane | 287 | 51.573 | 1.493 | 0.000105 | 0.0012 |
| GO:0030055 | cell-substrate junction | 411 | 73.856 | 1.3946 | 0.00015 | 0.001615 |
| GO:0044309 | neuron spine | 167 | 30.01 | 1.5995 | 0.000385 | 0.003897 |
| GO:0005802 | trans-Golgi network | 227 | 40.792 | 1.4954 | 0.000504 | 0.004815 |
| GO:0030427 | site of polarized growth | 166 | 29.83 | 1.5756 | 0.000635 | 0.005751 |
| GO:0043209 | myelin sheath | 158 | 28.392 | 1.5849 | 0.000715 | 0.006149 |
| GO:0048475 | coated membrane | 97 | 17.431 | 1.7211 | 0.001294 | 0.010601 |
| GO:0099572 | postsynaptic specialization | 340 | 61.097 | 1.3585 | 0.001479 | 0.011565 |
| GO:0043025 | neuronal cell body | 486 | 87.333 | 1.2824 | 0.002194 | 0.016409 |
| GO:0098984 | neuron to neuron synapse | 341 | 61.277 | 1.3382 | 0.002473 | 0.017721 |
| GO:0099568 | cytoplasmic region | 479 | 86.076 | 1.2779 | 0.002702 | 0.018122 |
| GO:0005770 | late endosome | 242 | 43.487 | 1.4027 | 0.002739 | 0.018122 |
| GO:0055037 | recycling endosome | 164 | 29.471 | 1.493 | 0.002965 | 0.018888 |
| GO:0005776 | autophagosome | 88 | 15.813 | 1.6442 | 0.005242 | 0.032203 |
| GO:0030667 | secretory granule membrane | 293 | 52.652 | 1.3295 | 0.005794 | 0.034365 |
| GO:0044306 | neuron projection terminus | 139 | 24.978 | 1.4813 | 0.006979 | 0.04001 |

**Supplementary Table 3.** Enriched GO terms (Molecular Functions) with false discovery date < 0.05 for Neuronal Essential Genes (NEGs).

| **Gene Set** | **Description** | **Size** | **Expect** | **Ratio** | **P Value** | **FDR** |
| --- | --- | --- | --- | --- | --- | --- |
| GO:0004674 | protein serine/threonine kinase activity | 449 | 80.53 | 2.2228 | 0 | 0 |
| GO:0000149 | SNARE binding | 102 | 18.294 | 2.4598 | 7.44E-10 | 1.05E-07 |
| GO:0044389 | ubiquitin-like protein ligase binding | 298 | 53.447 | 1.6278 | 9.90E-07 | 9.3E-05 |
| GO:0005543 | phospholipid binding | 412 | 73.894 | 1.5157 | 1.52E-06 | 0.000107 |
| GO:0000287 | magnesium ion binding | 211 | 37.844 | 1.7176 | 3.23E-06 | 0.000134 |
| GO:0003924 | GTPase activity | 293 | 52.551 | 1.5985 | 3.37E-06 | 0.000134 |
| GO:0004713 | protein tyrosine kinase activity | 178 | 31.925 | 1.7854 | 3.42E-06 | 0.000134 |
| GO:0019199 | transmembrane receptor protein kinase activity | 81 | 14.528 | 2.2027 | 3.99E-06 | 0.000134 |
| GO:0001882 | nucleoside binding | 384 | 68.872 | 1.51 | 4.29E-06 | 0.000134 |
| GO:0019001 | guanyl nucleotide binding | 390 | 69.948 | 1.5011 | 5.15E-06 | 0.000145 |
| GO:0048156 | tau protein binding | 45 | 8.0709 | 2.6019 | 8.71E-06 | 0.000223 |
| GO:0005484 | SNAP receptor activity | 30 | 5.3806 | 2.9736 | 1.23E-05 | 0.00029 |
| GO:0097472 | cyclin-dependent protein kinase activity | 37 | 6.6361 | 2.7124 | 1.87E-05 | 0.000381 |
| GO:0050321 | tau-protein kinase activity | 22 | 3.9458 | 3.2947 | 1.89E-05 | 0.000381 |
| GO:0051219 | phosphoprotein binding | 81 | 14.528 | 2.065 | 3.48E-05 | 0.000654 |
| GO:0019205 | nucleobase-containing compound kinase activity | 46 | 8.2503 | 2.4242 | 5.1E-05 | 0.000899 |
| GO:0004712 | protein serine/threonine/tyrosine kinase activity | 43 | 7.7122 | 2.4636 | 5.98E-05 | 0.000992 |
| GO:0016776 | phosphotransferase activity, phosphate group as acceptor | 38 | 6.8155 | 2.4943 | 0.000121 | 0.001888 |
| GO:0016853 | isomerase activity | 157 | 28.159 | 1.6691 | 0.000153 | 0.002271 |
| GO:0051117 | ATPase binding | 80 | 14.348 | 1.9514 | 0.000195 | 0.002748 |
| GO:0042578 | phosphoric ester hydrolase activity | 370 | 66.361 | 1.4014 | 0.000273 | 0.003661 |
| GO:0060589 | nucleoside-triphosphatase regulator activity | 348 | 62.415 | 1.4099 | 0.000318 | 0.004076 |
| GO:0030276 | clathrin binding | 57 | 10.223 | 2.0542 | 0.000546 | 0.006691 |
| GO:0030742 | GTP-dependent protein binding | 22 | 3.9458 | 2.7878 | 0.000606 | 0.007125 |
| GO:0051018 | protein kinase A binding | 48 | 8.609 | 2.0908 | 0.001049 | 0.011826 |
| GO:0017124 | SH3 domain binding | 126 | 22.599 | 1.6373 | 0.00109 | 0.011826 |
| GO:0046875 | ephrin receptor binding | 27 | 4.8426 | 2.478 | 0.001294 | 0.013512 |
| GO:0030145 | manganese ion binding | 61 | 10.941 | 1.9195 | 0.001503 | 0.015141 |
| GO:0035004 | phosphatidylinositol 3-kinase activity | 82 | 14.707 | 1.7679 | 0.001749 | 0.016826 |
| GO:0019838 | growth factor binding | 138 | 24.751 | 1.5757 | 0.00179 | 0.016826 |
| GO:0008022 | protein C-terminus binding | 187 | 33.539 | 1.461 | 0.002937 | 0.026715 |
| GO:0072341 | modified amino acid binding | 86 | 15.424 | 1.6856 | 0.00366 | 0.032253 |
| GO:0052813 | phosphatidylinositol bisphosphate kinase activity | 74 | 13.272 | 1.7329 | 0.004151 | 0.035468 |

**Supplementary Table 4.** Enriched GO terms (Biological Process) with false discovery date < 0.05 for Achilles Project-specific essential genes (ACEGs).

| **Gene Set** | **Description** | **Size** | **Expect** | **Ratio** | **P Value** | **FDR** |
| --- | --- | --- | --- | --- | --- | --- |
| GO:0006397 | mRNA processing | 487 | 37.527 | 4.1303 | 0 | 0 |
| GO:0044772 | mitotic cell cycle phase transition | 487 | 37.527 | 2.9046 | 0 | 0 |
| GO:0048285 | organelle fission | 459 | 35.37 | 2.6011 | 0 | 0 |
| GO:0022613 | ribonucleoprotein complex biogenesis | 440 | 33.906 | 5.5153 | 0 | 0 |
| GO:0008380 | RNA splicing | 417 | 32.133 | 4.2013 | 0 | 0 |
| GO:0006605 | protein targeting | 412 | 31.748 | 3.1183 | 0 | 0 |
| GO:0034470 | ncRNA processing | 367 | 28.28 | 4.9504 | 0 | 0 |
| GO:0051169 | nuclear transport | 356 | 27.433 | 3.1714 | 0 | 0 |
| GO:0006401 | RNA catabolic process | 341 | 26.277 | 4.2623 | 0 | 0 |
| GO:0033044 | regulation of chromosome organization | 329 | 25.352 | 2.9583 | 0 | 0 |
| GO:0090150 | establishment of protein localization to membrane | 313 | 24.119 | 3.2339 | 0 | 0 |
| GO:0007059 | chromosome segregation | 312 | 24.042 | 3.6186 | 0 | 0 |
| GO:0006260 | DNA replication | 268 | 20.652 | 3.8738 | 0 | 0 |
| GO:0071103 | DNA conformation change | 245 | 18.879 | 3.284 | 0 | 0 |
| GO:0071826 | ribonucleoprotein complex subunit organization | 245 | 18.879 | 4.926 | 0 | 0 |
| GO:0071824 | protein-DNA complex subunit organization | 242 | 18.648 | 3.9682 | 0 | 0 |
| GO:0016072 | rRNA metabolic process | 236 | 18.186 | 5.9937 | 0 | 0 |
| GO:0015931 | nucleobase-containing compound transport | 233 | 17.955 | 3.5646 | 0 | 0 |
| GO:0006403 | RNA localization | 228 | 17.569 | 4.4965 | 0 | 0 |
| GO:0006413 | translational initiation | 192 | 14.795 | 6.5562 | 0 | 0 |
| GO:0006399 | tRNA metabolic process | 183 | 14.102 | 4.4676 | 0 | 0 |
| GO:0140053 | mitochondrial gene expression | 159 | 12.252 | 4.8154 | 0 | 0 |
| GO:0031123 | RNA 3'-end processing | 141 | 10.865 | 4.5098 | 0 | 0 |
| GO:0070972 | protein localization to endoplasmic reticulum | 137 | 10.557 | 7.0096 | 0 | 0 |
| GO:0006414 | translational elongation | 133 | 10.249 | 5.0738 | 0 | 0 |
| GO:0071166 | ribonucleoprotein complex localization | 128 | 9.8634 | 5.272 | 0 | 0 |
| GO:0098781 | ncRNA transcription | 104 | 8.014 | 4.9912 | 0 | 0 |
| GO:0016073 | snRNA metabolic process | 95 | 7.3205 | 5.1909 | 0 | 0 |
| GO:0002181 | cytoplasmic translation | 92 | 7.0893 | 5.9244 | 0 | 0 |
| GO:0006353 | DNA-templated transcription, termination | 73 | 5.6252 | 6.3997 | 0 | 0 |
| GO:0090305 | nucleic acid phosphodiester bond hydrolysis | 288 | 22.193 | 3.019 | 1.11E-16 | 3.04E-15 |
| GO:1901987 | regulation of cell cycle phase transition | 386 | 29.744 | 2.656 | 4.44E-16 | 1.14E-14 |
| GO:0044843 | cell cycle G1/S phase transition | 247 | 19.033 | 3.1524 | 4.44E-16 | 1.14E-14 |
| GO:0032200 | telomere organization | 146 | 11.25 | 3.9109 | 9.99E-16 | 2.50E-14 |
| GO:0006360 | transcription by RNA polymerase I | 62 | 4.7776 | 5.8607 | 1.33E-15 | 3.24E-14 |
| GO:0006289 | nucleotide-excision repair | 110 | 8.4764 | 4.3651 | 3.89E-15 | 9.17E-14 |
| GO:0031503 | protein-containing complex localization | 280 | 21.576 | 2.8735 | 1.75E-14 | 4.03E-13 |
| GO:0034502 | protein localization to chromosome | 81 | 6.2417 | 4.8064 | 8.46E-14 | 1.86E-12 |
| GO:0006352 | DNA-templated transcription, initiation | 231 | 17.8 | 3.0336 | 8.55E-14 | 1.86E-12 |
| GO:0007051 | spindle organization | 175 | 13.485 | 3.337 | 2.84E-13 | 6.03E-12 |
| GO:0032984 | protein-containing complex disassembly | 323 | 24.89 | 2.6115 | 4.64E-13 | 9.63E-12 |
| GO:0042769 | DNA damage response, detection of DNA damage | 39 | 3.0053 | 6.655 | 7.76E-13 | 1.57E-11 |
| GO:1902850 | microtubule cytoskeleton organization involved in mitosis | 132 | 10.172 | 3.6376 | 2.26E-12 | 4.46E-11 |
| GO:1903311 | regulation of mRNA metabolic process | 266 | 20.497 | 2.732 | 2.98E-12 | 5.76E-11 |
| GO:0010608 | posttranscriptional regulation of gene expression | 486 | 37.45 | 2.1896 | 8.43E-12 | 1.59E-10 |
| GO:0006383 | transcription by RNA polymerase III | 55 | 4.2382 | 5.1909 | 2.93E-11 | 5.42E-10 |
| GO:0051383 | kinetochore organization | 21 | 1.6182 | 8.0335 | 3.59E-10 | 6.49E-09 |
| GO:0000075 | cell cycle checkpoint | 216 | 16.645 | 2.7036 | 5.94E-10 | 1.05E-08 |
| GO:0006354 | DNA-templated transcription, elongation | 98 | 7.5517 | 3.7078 | 6.56E-10 | 1.14E-08 |
| GO:0006302 | double-strand break repair | 218 | 16.799 | 2.6788 | 8.11E-10 | 1.34E-08 |
| GO:0036260 | RNA capping | 34 | 2.62 | 6.1069 | 8.14E-10 | 1.34E-08 |
| GO:0071897 | DNA biosynthetic process | 188 | 14.487 | 2.8301 | 8.17E-10 | 1.34E-08 |
| GO:0051052 | regulation of DNA metabolic process | 405 | 31.209 | 2.1468 | 1.69E-09 | 2.71E-08 |
| GO:0010948 | negative regulation of cell cycle process | 278 | 21.422 | 2.3807 | 4.61E-09 | 7.26E-08 |
| GO:0006333 | chromatin assembly or disassembly | 155 | 11.944 | 2.8466 | 1.93E-08 | 2.99E-07 |
| GO:0031023 | microtubule organizing center organization | 134 | 10.326 | 3.0022 | 2.19E-08 | 3.32E-07 |
| GO:0045787 | positive regulation of cell cycle | 374 | 28.82 | 2.0819 | 3.85E-08 | 5.75E-07 |
| GO:0022616 | DNA strand elongation | 24 | 1.8494 | 6.4886 | 4.69E-08 | 6.87E-07 |
| GO:0050000 | chromosome localization | 78 | 6.0105 | 3.6602 | 5.72E-08 | 8.24E-07 |
| GO:0006338 | chromatin remodeling | 150 | 11.559 | 2.7685 | 1.00E-07 | 1.42E-06 |
| GO:0044839 | cell cycle G2/M phase transition | 213 | 16.413 | 2.437 | 1.10E-07 | 1.54E-06 |
| GO:0006301 | postreplication repair | 51 | 3.93 | 4.3257 | 1.26E-07 | 1.73E-06 |
| GO:0045930 | negative regulation of mitotic cell cycle | 256 | 19.727 | 2.2812 | 1.37E-07 | 1.85E-06 |
| GO:0006310 | DNA recombination | 273 | 21.037 | 2.1391 | 9.01E-07 | 1.2E-05 |
| GO:0034248 | regulation of cellular amide metabolic process | 385 | 29.667 | 1.9213 | 1.28E-06 | 1.67E-05 |
| GO:0034504 | protein localization to nucleus | 267 | 20.575 | 2.09 | 2.93E-06 | 3.78E-05 |
| GO:0072331 | signal transduction by p53 class mediator | 218 | 16.799 | 2.2026 | 4.13E-06 | 5.24E-05 |
| GO:0009451 | RNA modification | 152 | 11.713 | 2.4759 | 4.34E-06 | 5.43E-05 |
| GO:0008334 | histone mRNA metabolic process | 29 | 2.2347 | 4.9224 | 5.13E-06 | 6.31E-05 |
| GO:0060249 | anatomical structure homeostasis | 408 | 31.44 | 1.813 | 7.94E-06 | 9.64E-05 |
| GO:0043631 | RNA polyadenylation | 48 | 3.6988 | 3.785 | 9.62E-06 | 0.000115 |
| GO:0061641 | CENP-A containing chromatin organization | 31 | 2.3888 | 4.6048 | 1.09E-05 | 0.000128 |
| GO:0017038 | protein import | 211 | 16.259 | 2.1526 | 1.25E-05 | 0.000145 |
| GO:0006997 | nucleus organization | 126 | 9.7093 | 2.4719 | 2.92E-05 | 0.000336 |
| GO:0016569 | covalent chromatin modification | 468 | 36.063 | 1.6915 | 3.35E-05 | 0.00038 |
| GO:0000959 | mitochondrial RNA metabolic process | 41 | 3.1594 | 3.7982 | 4E-05 | 0.000447 |
| GO:0006490 | oligosaccharide-lipid intermediate biosynthetic process | 20 | 1.5412 | 5.1909 | 6.6E-05 | 0.000725 |
| GO:0018205 | peptidyl-lysine modification | 360 | 27.741 | 1.7663 | 6.65E-05 | 0.000725 |
| GO:0010639 | negative regulation of organelle organization | 371 | 28.589 | 1.749 | 7.26E-05 | 0.000781 |
| GO:0000910 | cytokinesis | 159 | 12.252 | 2.2037 | 7.89E-05 | 0.000838 |
| GO:0040029 | regulation of gene expression, epigenetic | 258 | 19.881 | 1.9114 | 8.21E-05 | 0.000861 |
| GO:0070646 | protein modification by small protein removal | 288 | 22.193 | 1.8475 | 9.52E-05 | 0.000987 |
| GO:0031163 | metallo-sulfur cluster assembly | 21 | 1.6182 | 4.9437 | 9.94E-05 | 0.001018 |
| GO:1902579 | multi-organism localization | 33 | 2.5429 | 3.9325 | 0.000127 | 0.001287 |
| GO:1903320 | regulation of protein modification by small protein conjugation or removal | 217 | 16.722 | 1.9137 | 0.000284 | 0.002838 |
| GO:0006284 | base-excision repair | 43 | 3.3135 | 3.3198 | 0.000313 | 0.003092 |
| GO:0051321 | meiotic cell cycle | 248 | 19.11 | 1.8315 | 0.000355 | 0.003468 |
| GO:0032259 | methylation | 346 | 26.662 | 1.6878 | 0.000366 | 0.003537 |
| GO:2001020 | regulation of response to DNA damage stimulus | 203 | 15.643 | 1.9178 | 0.000418 | 0.00399 |
| GO:0006513 | protein monoubiquitination | 67 | 5.1629 | 2.7117 | 0.000492 | 0.004643 |
| GO:0032069 | regulation of nuclease activity | 22 | 1.6953 | 4.1291 | 0.000963 | 0.009 |
| GO:0000726 | non-recombinational repair | 81 | 6.2417 | 2.4032 | 0.00119 | 0.010877 |
| GO:0071806 | protein transmembrane transport | 65 | 5.0088 | 2.5954 | 0.00119 | 0.010877 |
| GO:0036297 | interstrand cross-link repair | 50 | 3.8529 | 2.855 | 0.001235 | 0.011167 |
| GO:0031647 | regulation of protein stability | 267 | 20.575 | 1.6525 | 0.002582 | 0.02307 |
| GO:0033108 | mitochondrial respiratory chain complex assembly | 96 | 7.3976 | 2.1629 | 0.002606 | 0.02307 |
| GO:0031109 | microtubule polymerization or depolymerization | 108 | 8.3223 | 2.0427 | 0.003613 | 0.031486 |
| GO:0001701 | in utero embryonic development | 345 | 26.585 | 1.5422 | 0.00363 | 0.031486 |
| GO:0051656 | establishment of organelle localization | 494 | 38.067 | 1.4186 | 0.005645 | 0.048464 |

**Supplementary Table 5.** Enriched GO terms (Cellular Component) with false discovery date < 0.05 for Achilles Project-specific essential genes (ACEGs).

| **Gene Set** | **Description** | **Size** | **Expect** | **Ratio** | **P Value** | **FDR** |
| --- | --- | --- | --- | --- | --- | --- |
| GO:0098687 | chromosomal region | 331 | 30.282 | 3.2362 | 0 | 0 |
| GO:0044445 | cytosolic part | 244 | 22.323 | 3.7182 | 0 | 0 |
| GO:0005840 | ribosome | 229 | 20.95 | 5.4892 | 0 | 0 |
| GO:0005681 | spliceosomal complex | 176 | 16.102 | 3.9127 | 0 | 0 |
| GO:0030684 | preribosome | 73 | 6.6785 | 5.091 | 0 | 0 |
| GO:0120114 | Sm-like protein family complex | 75 | 6.8615 | 4.8095 | 1.44E-15 | 4.14E-14 |
| GO:0000793 | condensed chromosome | 223 | 20.401 | 2.8429 | 8.79E-14 | 2.16E-12 |
| GO:0098798 | mitochondrial protein complex | 266 | 24.335 | 2.4655 | 2.38E-11 | 5.12E-10 |
| GO:0032993 | protein-DNA complex | 163 | 14.912 | 2.8165 | 3.41E-10 | 6.52E-09 |
| GO:0034708 | methyltransferase complex | 116 | 10.612 | 3.2038 | 4.24E-10 | 7.29E-09 |
| GO:0044452 | nucleolar part | 178 | 16.285 | 2.7019 | 5.48E-10 | 8.57E-09 |
| GO:0005657 | replication fork | 69 | 6.3126 | 3.9604 | 6.46E-10 | 9.27E-09 |
| GO:0061695 | transferase complex, transferring phosphorus-containing groups | 254 | 23.238 | 2.3669 | 8.38E-10 | 1.11E-08 |
| GO:0005759 | mitochondrial matrix | 462 | 42.267 | 1.9637 | 9.22E-10 | 1.13E-08 |
| GO:0005844 | polysome | 73 | 6.6785 | 3.7434 | 2.53E-09 | 2.90E-08 |
| GO:0005819 | spindle | 328 | 30.008 | 2.1328 | 3.11E-09 | 3.34E-08 |
| GO:0005743 | mitochondrial inner membrane | 455 | 41.626 | 1.9219 | 5.30E-09 | 5.36E-08 |
| GO:0005732 | small nucleolar ribonucleoprotein complex | 21 | 1.9212 | 6.246 | 4.33E-08 | 4.14E-07 |
| GO:0016607 | nuclear speck | 383 | 35.039 | 1.9121 | 1.21E-07 | 1.09E-06 |
| GO:1905348 | endonuclease complex | 29 | 2.6531 | 4.523 | 3.82E-06 | 3.29E-05 |
| GO:0015030 | Cajal body | 55 | 5.0318 | 3.3785 | 4.57E-06 | 3.74E-05 |
| GO:0044815 | DNA packaging complex | 75 | 6.8615 | 2.9148 | 8.45E-06 | 6.6E-05 |
| GO:1902493 | acetyltransferase complex | 92 | 8.4167 | 2.6138 | 2.03E-05 | 0.000152 |
| GO:0005697 | telomerase holoenzyme complex | 20 | 1.8297 | 4.9188 | 2.86E-05 | 0.000197 |
| GO:0035145 | exon-exon junction complex | 20 | 1.8297 | 4.9188 | 2.86E-05 | 0.000197 |
| GO:0005849 | mRNA cleavage factor complex | 21 | 1.9212 | 4.6845 | 4.61E-05 | 0.000305 |
| GO:0030055 | cell-substrate junction | 411 | 37.601 | 1.6489 | 4.97E-05 | 0.000316 |
| GO:0099023 | tethering complex | 67 | 6.1296 | 2.6103 | 0.000272 | 0.001669 |
| GO:0000790 | nuclear chromatin | 341 | 31.197 | 1.6348 | 0.000283 | 0.001681 |
| GO:0016592 | mediator complex | 35 | 3.202 | 3.123 | 0.000858 | 0.004921 |
| GO:0034399 | nuclear periphery | 133 | 12.168 | 1.9724 | 0.000902 | 0.005004 |
| GO:0090734 | site of DNA damage | 66 | 6.0381 | 2.3186 | 0.002205 | 0.011852 |
| GO:1905368 | peptidase complex | 89 | 8.1423 | 2.0879 | 0.002605 | 0.01358 |
| GO:0035770 | ribonucleoprotein granule | 214 | 19.578 | 1.6345 | 0.00367 | 0.018566 |
| GO:0005875 | microtubule associated complex | 148 | 13.54 | 1.7725 | 0.003968 | 0.0195 |
| GO:1905354 | exoribonuclease complex | 26 | 2.3786 | 2.9428 | 0.007313 | 0.034938 |
| GO:0070469 | respiratory chain | 100 | 9.1486 | 1.8582 | 0.008837 | 0.041082 |
| GO:0000151 | ubiquitin ligase complex | 273 | 24.976 | 1.4814 | 0.009656 | 0.043708 |
| GO:0070069 | cytochrome complex | 34 | 3.1105 | 2.5719 | 0.01005 | 0.044324 |

**Supplementary Table 6.** Enriched GO terms (Molecular Functions) with false discovery date < 0.05 for Achilles Project-specific essential genes (ACEGs).

| **Gene Set** | **Description** | **Size** | **Expect** | **Ratio** | **P Value** | **FDR** |
| --- | --- | --- | --- | --- | --- | --- |
| GO:0140098 | catalytic activity, acting on RNA | 350 | 26.274 | 4.0724 | 0 | 0 |
| GO:0003735 | structural constituent of ribosome | 156 | 11.711 | 8.283 | 0 | 0 |
| GO:0016779 | nucleotidyltransferase activity | 129 | 9.6839 | 4.3371 | 0 | 0 |
| GO:0019843 | rRNA binding | 65 | 4.8795 | 5.7383 | 2.89E-15 | 2.04E-13 |
| GO:0004386 | helicase activity | 150 | 11.26 | 3.5523 | 6.78E-13 | 3.82E-11 |
| GO:0003729 | mRNA binding | 233 | 17.491 | 2.8586 | 7.36E-12 | 3.46E-10 |
| GO:0008135 | translation factor activity, RNA binding | 87 | 6.531 | 4.1341 | 8.89E-11 | 3.58E-09 |
| GO:0000049 | tRNA binding | 59 | 4.4291 | 4.5156 | 4.41E-09 | 1.48E-07 |
| GO:0016874 | ligase activity | 151 | 11.335 | 2.9994 | 4.71E-09 | 1.48E-07 |
| GO:0003697 | single-stranded DNA binding | 107 | 8.0324 | 3.3614 | 1.40E-08 | 3.95E-07 |
| GO:0043021 | ribonucleoprotein complex binding | 129 | 9.6839 | 2.9947 | 6.62E-08 | 1.70E-06 |
| GO:0140097 | catalytic activity, acting on DNA | 184 | 13.813 | 2.5339 | 2.54E-07 | 5.97E-06 |
| GO:0016887 | ATPase activity | 438 | 32.88 | 1.916 | 3.62E-07 | 7.86E-06 |
| GO:0017069 | snRNA binding | 39 | 2.9277 | 4.4404 | 2.85E-06 | 5.75E-05 |
| GO:0031369 | translation initiation factor binding | 31 | 2.3271 | 4.7268 | 8.40E-06 | 0.000158 |
| GO:0004518 | nuclease activity | 205 | 15.389 | 2.2094 | 9.25E-06 | 0.000163 |
| GO:0042162 | telomeric DNA binding | 38 | 2.8526 | 4.2067 | 1.3E-05 | 0.000215 |
| GO:0036002 | pre-mRNA binding | 33 | 2.4773 | 4.4404 | 1.67E-05 | 0.000262 |
| GO:0051082 | unfolded protein binding | 118 | 8.8581 | 2.5965 | 1.88E-05 | 0.000279 |
| GO:0003713 | transcription coactivator activity | 316 | 23.722 | 1.897 | 2.22E-05 | 0.000313 |
| GO:0003684 | damaged DNA binding | 69 | 5.1798 | 3.0889 | 3.87E-05 | 0.000498 |
| GO:0042393 | histone binding | 192 | 14.413 | 2.1508 | 3.88E-05 | 0.000498 |
| GO:0030515 | snoRNA binding | 31 | 2.3271 | 4.2971 | 5.59E-05 | 0.000686 |
| GO:0017056 | structural constituent of nuclear pore | 26 | 1.9518 | 4.6111 | 7.07E-05 | 0.000831 |
| GO:0017025 | TBP-class protein binding | 22 | 1.6515 | 4.844 | 0.000121 | 0.001363 |
| GO:0051540 | metal cluster binding | 62 | 4.6543 | 3.008 | 0.000158 | 0.001715 |
| GO:0001098 | basal transcription machinery binding | 53 | 3.9787 | 3.0161 | 0.000448 | 0.004678 |
| GO:0003727 | single-stranded RNA binding | 88 | 6.6061 | 2.422 | 0.000763 | 0.007681 |
| GO:0008565 | protein transporter activity | 89 | 6.6811 | 2.3948 | 0.000866 | 0.008185 |
| GO:0140104 | molecular carrier activity | 42 | 3.1529 | 3.1717 | 0.000871 | 0.008185 |
| GO:0070491 | repressing transcription factor binding | 73 | 5.48 | 2.5547 | 0.000929 | 0.008449 |
| GO:0031491 | nucleosome binding | 68 | 5.1047 | 2.5467 | 0.001441 | 0.012696 |
| GO:0001047 | core promoter binding | 45 | 3.3781 | 2.9602 | 0.001538 | 0.01314 |
| GO:0046982 | protein heterodimerization activity | 468 | 35.132 | 1.5086 | 0.001642 | 0.013436 |
| GO:0044389 | ubiquitin-like protein ligase binding | 298 | 22.371 | 1.654 | 0.001668 | 0.013436 |
| GO:0016741 | transferase activity, transferring one-carbon groups | 221 | 16.59 | 1.748 | 0.002242 | 0.017561 |
| GO:0047485 | protein N-terminus binding | 104 | 7.8072 | 2.0494 | 0.004536 | 0.034573 |

**Supplementary Table 7.** Differential expression (DE) among controls and neurological disorders in brain tissue transcriptome data (results from Figure 2).

| **Dataset** | **Disease** | **Direction** | **Wilcoxon rank sum test p-value** | **Cohen's D** | **95 percent confidence interval** |
| --- | --- | --- | --- | --- | --- |
| GSE95587 | Alzheimer's disease | Down | 8.969E-11 | 0.2986 | -0.4894809 -0.2429025 |
|  |  | Up | 5.173E-01 | 0.0267 | -0.14575432 -0.07121057 |
| GSE64018 | Autism spectrum disorder | Down | 2.239E-07 | 0.2755 | -0.2408972 -0.1032480 |
|  |  | Up | 2.864E-01 | -0.0063 | -0.07430813 0.02101093 |
| GSE64810 | Huntington's Diseases | Down | 3.415E-03 | 0.0811 | -0.16542582 -0.03102544 |
|  |  | Up | 4.772E-09 | 0.267 | -0.3343027 -0.1516005 |
| GSE122649 | Amyotrophic lateral sclerosis | Down | 3.030E-03 | 0.112 | -0.12708766 -0.02377154 |
|  |  | Up | 9.870E-01 | -0.0214 | -0.04331088 0.04321161 |
| GSE68719 | Parkinson's disease | Down | 4.864E-01 | 0.1041 | -0.05127025 0.11134400 |
|  |  | Up | 2.392E-01 | -0.0401 | -0.14412535 0.03295192 |
| CommondMind | Schizophrenia | Down | 2.755E-02 | 0.1312 | -0.028167711 -0.001331744 |
|  |  | Up | 1.217E-03 | 0.2269 | -0.031309724 -0.006582668 |

**Supplementary Table 8.** Summary of the GWAS and *de novo* mutation data.

| **Dataset** | **Variant Type** | **Description** | **Patients (n)** | **Control Subjects (n)** | **Analysis program** |
| --- | --- | --- | --- | --- | --- |
| **Psychiatric Disorders** | | | | | |
| **Schizophrenia (SCZ)** | | | | | |
| SCZ GWAS | Common | PGC + deCODE meta-analysis | 36989 | 113075 | MAGMA |
| SCZ Rare | Rare | Rare variants (MAF <1%) | 5585 | 8103 | MAGMA |
| SCZ Ultra-rare | Ultra-rare | 43 genes enriched for deleterious ultra-rare variants (p < .01) | 4877 | 6242 | Fisher’s exact test |
| SCZ DNM LOF | De novo | denovo-db | 300 | NA | denovolyzeR |
| **Bipolar Disorder (BD)** | | | | | |
| BD GWAS | Common | PGC | 3548 | 8965 | MAGMA |
| BD Rare | Rare | Rare variants (MAF <1%) | 926 | 1719 | MAGMA |
| BD DNM LOF | De novo | denovo-db | 42 |  | denovolyzeR |
| BD DNM Protein-Altering | De novo | denovo-db | 42 |  | denovolyzeR |
| **Neurodegenerative disease** | | | | | |
| **Autism Spectrum Disorder (ASD)** | | | | | |
| ASD iPSYCH-PGC | Common | iPSYCH + PGC meta-analysis | 18381 | 27969 | MAGMA |
| ASD SFARI | Gene list | 80 literature-curated genes | NA | NA | Fisher’s exact test |
| ASD Sanders | Rare | 65 genes (FDR < .1) | 3982 | 1911 | Fisher’s exact test |
| ASD DNM LOF | De novo | denovo-db | 4424 | NA | denovolyzeR |
| **Alzheimer Disease (AD)** | | | | | |
| AD GWAS | Common | GWAS Catalog | 14338 | 27696 | MAGMA |
| AD DNM Protein-Altering | De novo | denovo-db | 7 |  | denovolyzeR |
| **Parkinson's Disease (PD)** | | | | | |
| PD GWAS | Common | GWAS Catalog | 6476 | 30242 | MAGMA |
| PD DNM Protein-Altering | De novo | denovo-db | 15 |  | denovolyzeR |
| **Amyotrophic Lateral Sclerosis** | | | | | |
| ALS GWAS | Common | GWAS Catalog | 2579 | 2767 | MAGMA |
| ALS DNM lof | De novo | denovo-db | 79 |  | denovolyzeR |
| ALS DNM Protein-Altering | De novo | denovo-db | 79 |  | denovolyzeR |
| **Neurodevelopmental disorders** | | | | | |
| **Developmental Disorder (DD)** | | | | | |
| DDG2P(Developmental Disorder Gene2Phenotype) | Gene list | DDG2P | 14000 |  | Fisher’s exact test |
| **Intellectual Disability (ID)** | | | | | |
| ID risk genes | Gene list |  | 3032 | 4058 | Fisher’s exact test |
| ID DNM | De novo | denovo-db |  |  | denovolyzeR |
| **Epilepsy(EPI)** | | | | | |
| EPI risk genes | Gene list |  | 2127 | 4689 | Fisher’s exact test |
| EPI DNM LOF | De novo | denovo-db | 278 |  | denovolyzeR |
| EPI DNM Protein-Altering | De novo | denovo-db | 278 |  | denovolyzeR |
| **Cerebral palsy (CP)** | | | | | |
| CP DNM LOF | De novo | denovo-db | 43 |  | denovolyzeR |
| CP DNM Protein-Altering | De novo | denovo-db | 43 |  | denovolyzeR |
